# Supplementary material for: Novel approaches in linkage of data sources to explore the associations between purchase of opioid prescriptions during pregnancy and adverse neonatal outcomes
Source: PLoS One. 2026 Jan 30;21(1):e0340816. doi: 10.1371/journal.pone.0340816 (PMC12857999; doi:10.1371/journal.pone.0340816)
Supplement: S1 Appendix — (DOCX) [file pone.0340816.s001.docx]

**S1 Appendix**. Data sources and definitions of exposure, outcome, and covariate Measures.

| **Main Exposure (OpiAte Analgesic DRUG NAME)** | **DEFINITION** | | **Source** |
| --- | --- | --- | --- |
| Butorphanol | All prescribed opioids analgesic were identified using the National Drug Codes, allowing the calculation of morphine milligram equivalents (MME).^a^ | | APCD maternal pharmacy claims and PDMP |
| Codeine |  |  |  |
| Dihydrocodeine |  |  |  |
| Fentanyl long-acting |  |  |  |
| Fentanyl short-acting |  |  |  |
| Hydrocodone long-acting |  |  |  |
| Hydrocodone short-acting |  |  |  |
| Hydromorphone long-acting |  |  |  |
| Hydromorphone short-acting |  |  |  |
| Levomethadyl long-acting |  |  |  |
| Levorphanol long-acting |  |  |  |
| Meperidine short-acting |  |  |  |
| Methadone long-acting |  |  |  |
| Morphine long-acting |  |  |  |
| Morphine short-acting |  |  |  |
| Opium short-acting |  |  |  |
| Oxycodone long-acting |  |  |  |
| Oxycodone short-acting |  |  |  |
| Oxymorphone long-acting |  |  |  |
| Oxymorphone short-acting |  |  |  |
| Pentazocine |  |  |  |
| Propoxyphene |  |  |  |
| Tapentadol long-acting |  |  |  |
| Tapentadol short-acting |  |  |  |
| Tramadol long-acting |  |  |  |
| Tramadol short-acting |  |  |  |
|  |  | |  |
| **Outcome Measure** | **Definition** | | **Source** |
| Preterm delivery^b^ | Gestational age at birth <37 weeks  PPV: Varied between  83% (95% CI: 79%, 86%) to  94% (95% CI: 92%, 96%) based on method of estimation of gestational age | | Gestational age extracted from BCR |
| Low birth weight^b^ | Birth weight <2500 g  PPV: 99% (96%, 100%) | | Birth weight extracted from BCR |
| Small for gestational age | Birth weight below the mean Z-score of birth weight per sex and gestational age based on the U.S. National Growth Curve^c^ | | Birth weight, gestational age, and sex of infant extracted from BCR |
| Neonatal intensive care unit admission^b^ | NICU admission following birth  PPV: 93% (87%, 99%) | | Variable extracted from BCR |
| Neonatal opioid withdrawal syndrome^d^ | ICD-9-CM: 779.5-PPV: 91% (95% CI: 88.8%–92.5%)  ICD-10-CM: P961-PPV: 98.2% (95% CI: 95.4%–99.2%) | | APCD neonatal medical claims |
|  |  | |  |
| **Variables** | **Definition** | | **Source** |
| *Maternal demographic characteristics* |  | | |
| Maternal age group | <20 years  ≥20−<30 years  ≥30 years | | BCR |
| Race/ethnicity | Non-Hispanic White  Non-Hispanic Black  Other | | BCR |
| Marital status | (yes/no) | | BCR |
| Prenatal care | (yes/no) | | BCR |
| Eligibility for WIC | (yes/no) | | BCR |
| Pre-pregnancy BMI | ≤18  19−24  25−29  ≥30 | | Based on pre-pregnancy weight and height from BCR |
| Geographic location of maternal residence | North  Central  South | | Based on 5-digit zip code of maternal address at time of delivery extracted from BCR |
| Level of education | Less than high school  High school  More than high school  Missing | | BCR |
| Payer type | Medicaid  Private insurance  Other | | BCR |
|  |  | |  |
| *Rurality of maternal area of residence^e^* | Metropolitan (2010 RUCA codes): 1-3  Micropolitan (2010 RUCA codes): 4-6  Small (2010 RUCA codes): 7-9  Rural (2010 RUCA codes): 10 | | Based on linking the 5-digit zip code of maternal address at time of delivery extracted from BCR to the 2010 RUCA codes extracted from the SDOH database |
|  |  | |  |
| *Maternal medical diagnosis* | ICD-9-CM Codes | ICD-10-CM Codes | APCD maternal medical claims |
| Mental health disorders^f^ |  |  |  |
| 1. Depressive disorders | 311, 2960x, 2961x, 2962x, 2963x, 2964x, | F320, F321 F322, F323, F324, F325, F328, F329, F330, F331, F332, F333, F3340, F3341, F3342, F338, F339 |  |
| 1. Anxiety disorders | 29384, 30000, 30001, 30002, 30009, 30010, 30020, 30021, 30022, 30023, 30029, 3003, 3005, 30089, 3009, 3080, 3081, 3082, 3084, 3089, 30921, 3130, 3131, 31321, 31322, 3133, 31382, 31383 | F064, F4000, F4001, F4002, F4010, F4011, F40210, F40218, F40220, F40228, F40230, F40231, F40232, F40233, F40240, F40241, F40242, F40243, F40248, F40290, F40291, F40298, F408, F409, F410, F411, F413, F418, F419, F42, F430, F4311, F4312, F488, F489, R452, R453, R454, R455, R456, R457, R4581, R4582, R4583, R4584, F930 |  |
| 1. Conduct disorders | 31200, 31201, 31202, 31203, 31210, 31211, 31212, 31213, 31220, 31221, 31222, 31223, 3124, 3128, 31281, 31282, 31289, 3129 | F910, F911, F912, F918, F919 |  |
| 1. Bipolar disorders | 29600, 29601, 29602, 29603, 29604, 29605, 29606, 29610, 29611, 29612, 29613, 29614, 29615, 29616, 29640, 29641, 29642 29643, 29644, 29645, 29646, 29650, 29651, 29652, 29653, 29654, 29655, 29656, 29660, 29661, 29662, 29663, 29664, 29665, 29666, 2967, 29680, 29681, 29682, 29689, 29690, 29699 | F310, F3110, F3111, F3112, F3113, F312, F3130, F3131, F3132, F314, F315, F3160, F3161, F3162, F3163, F3164, F3170, F3171, F3172, F3173, F3174, F3175, F3176, F3177, F3178, F3181, F3189, F319 |  |
| 1. Trauma and stressor related disorders | 3083, 3090, 3091, 30922, 30923, 30924, 30928, 30929, 3093, 3094, 30981, 30982, 30983, 30989, 3099 | F4310, F4320, F4321, F4322, F4323, F4324, F4325, F4329, F438, F439 |  |
| 1. Schizophrenia and other psychotic disorders | 29381, 29382, 29500, 29501, 29502, 29503, 29504, 29505, 29510, 29511, 29512, 29513, 29514, 29515, 29520, 29521, 29522, 29523, 29524, 29525, 29530, 29531, 29532, 29533, 29534, 29535, 29540, 29541, 29542, 29543, 29544, 29545, 29550, 29551, 29552, 29553, 29554, 29555, 29560, 29561, 29562, 29563, 29564, 29565, 29570, 29571, 29572, 29573, 29574, 29575, 29580, 29581, 29582, 29583, 29584, 29585, 29590, 29591, 29592, 29593, 29594, 29595, 2970, 2971, 2972, 2973, 2978, 2979, 2980, 2981, 2982, 2983, 2984, 2988, 2989 | F060, F062, F200, F201, F202, F203, F205, F2081, F2089, F209, F21, F22, F23, F24, F250, F251, F258, F259, F28, F29 |  |
| 1. Personality disorders | 3010, 30110, 30111, 30112, 30113, 30120, 30121, 30122, 3013, 3014, 30150, 30151, 30159, 3016, 3017, 30181, 30182, 30183, 30184, 30189, 3019 | F600, F601, F602, F603, F604, F605, F606, F607, F6081, F6089, F609, F69 |  |
| 1. Other mental health diagnoses | 30012, 30013, 30014, 30015, 3006, 3071, 30750, 30751, 30752, 30753, 30754, 30759, 30016, 30019, 3060, 3061, 3062, 3063, 3064, 30650, 30652, 30653, 30659, 3066, 3067, 3068, 3069, 3021, 3022, 3023, 3024, 30250, 30251, 30252, 30253, 3026, 30270, 30271, 30272, 30273, 30274, 30275, 30276, 30279, 30281, 30282, 30283, 30284, 30285, 30289, 3029, 30651, 30740, 30741, 30742, 30743, 30744, 30745, 30746, 30747, 30748, 30749, 30011, 3007, 30081, 30082, 30780, 30781, 30789, 29389, 2939, 3101, 316, 64840, 64841, 64842, 64843, 64844, V402, V403, V4031, V4039, V409, V673 | F061, F068, F440, F441, F442, F444, F445, F446, F447, F4481, F4489, F449, F450, F451, F4520, F4521, F4522, F4529, F4541, F4542, F458, F459, F481, F5000, F5001, F5002, F502, F508, F509, F5101, F5102, F5103, F5104, F5105, F5109, F5111, F5112, F5113, F5119, F513, F514, F515, F518, F519, F520, F521, F5221, F5222, F5231, F5232, F524, F525, F526, F528, F529, F53, F54, F59, F641, F648, F649, F650, F651, F652, F653, F654, F6550, F6551, F6552, F6581, F6589, F659, F66, F6810, F6811, F6812, F6813, F688, F99, R37, R4589, R630, R632, Z87890, Z9183, O9934^g^ |  |
| Substances use disorder^f^ | 2920, 29211, 29212, 2922, 29281, 29282, 29283, 29284, 29285, 29289, 2929, 30400, 30401, 30402, 30403, 30410, 30411, 30412, 30413, 30420, 30421, 30422, 30423, 30430, 30431, 30432, 30433, 30440, 30441, 30442, 30443, 30450, 30451, 30452, 30453, 30460, 30461, 30462, 30463, 30470, 30471, 30472, 30473, 30480, 30481, 30482, 30483, 30490, 30491, 30492, 30493, 30520, 30521, 30522, 30523, 30530, 30531, 30532, 30533, 30540, 30541, 30542, 30543, 30550, 30551, 30552, 30553, 30560, 30561, 30562, 30563, 30570, 30571, 30572, 30573, 30580, 30581, 30582, 30583, 30590, 30591, 30592, 30593, 64830, 64831, 64832, 64833, 64834, 65550, 65551, 65553, 76072, 76073, 76075, 7795, 96500, 96501, 96502, 96509, V6542 | F1110, F11120, F11121, F11122, F11129, F1114, F11150, F11151, F11159, F11181, F11182, F11188, F1119, F1120, F1121, F11220, F11221, F11222, F11229, F1123, F1124, F11250, F11251, F11259, F11281, F11282, F11288, F1129, F1190, F11920, F11921, F11922, F11929, F1193, F1194, F11950, F11951, F11959, F11981, F11982, F11988, F1199, F1210, F12120, F12121, F12122, F12129, F12150, F12151, F12159, F12180, F12188, F1219, F1220, F1221, F12220, F12221, F12222, F12229, F12250, F12251, F12259, F12280, F12288, F1229, F1290, F12920, F12921, F12922, F12929, F12950, F12951, F12959, F12980, F12988, F1299, F1310, F13120, F13121, F13129, F1314, F13150, F13151, F13159, F13180, F13181, F13182, F13188, F1319, F1320, F1321, F13220, F13221, F13229, F13230, F13231, F13232, F13239, F1324, F13250, F13251, F13259, F1326, F1327, F13280, F13281, F13282, F13288, F1329, F1390, F13920, F13921, F13929, F13930, F13931, F13932, F13939, F1394, F13950, F13951, F13959, F1396, F1397, F13980, F13981, F13982, F13988, F1399, F1410, F14120, F14121, F14122, F14129, F1414, F14150, F14151, F14159, F14180, F14181, F14182, F14188, F1419, F1420, F1421, F14220, F14221, F14222, F14229, F1423, F1424, F14250, F14251, F14259, F14280, F14281, F14282, F14288, F1429, F1490, F14920, F14921, F14922, F14929, F1494, F14950, F14951, F14959, F14980, F14981, F14982, F14988, F1499, F1510, F15120, F15121, F15122, F15129, F1514, F15150, F15151, F15159, F15180, F15181, F15182, F15188, F1519, F1520, F1521, F15220, F15221, F15222, F15229, F1523, F1524, F15250, F15251, F15259, F15280, F15281, F15282, F15288, F1529, F1590, F15920, F15921, F15922, F15929, F1593, F1594, F15950, F15951, F15959, F15980, F15981, F15982, F15988, F1599, F1610, F16120, F16121, F16122, F16129, F1614, F16150, F16151, F16159, F16180, F16183, F16188, F1619, F1620, F1621, F16220, F16221, F16229, F1624, F16250, F16251, F16259, F16280, F16283, F16288, F1629, F1690, F16920, F16921, F16929, F1694, F16950, F16951, F16959, F16980, F16983, F16988, F1699, F17200, F17201, F17203, F17208, F17209, F17210, F17211, F17213, F17218, F17219, F17220, F17221, F17223, F17228, F17229, F17290, F17291, F17293, F17298, F17299, F1810, F18120, F18121, F18129, F1814, F18150, F18151, F18159, F1817, F18180, F18188, F1819, F1820, F1821, F18220, F18221, F18229, F1824, F18250, F18251, F18259, F1827, F18280, F18288, F1829, F1890, F18920, F18921, F18929, F1894, F18950, F18951, F18959, F1897, F18980, F18988, F1899, F1910, F19120, F19121, F19122, F19129, F1914, F19150, F19151, F19159, F1916, F1917, F19180, F19181, F19182, F19188, F1919, F1920, F1921, F19220, F19221, F19222, F19229, F19230, F19231, F19232, F19239, F1924, F19250, F19251, F19259, F1926, F1927, F19280, F19281, F19282, F19288, F1929, F1990, F19920, F19921, F19922, F19929, F19930, F19931, F19932, F19939, F1994, F19950, F19951, F19959, F1996, F1997, F19980, F19981, F19982, F19988, F1999, F550, F551, F552, F553, F554, F558, O355XX0,^g^ O355XX1,^g^ O355XX2,^g^ O355XX3,^g^ O355XX4,^g^ O355XX5,^g^ O355XX9,^g^ O99320,^g^ O99321,^g^ O99322,^g^ O99323,^g^ O99324,^g^ O99325,^g^ P0441, P0449, P961, P962, T400X1A, T400X1D, T400X1S, T400X3A, T400X3D, T400X3S, T400X4A, T400X4D, T400X4S, T400X5A, T400X5D, T400X5S, T400X6A, T400X6D, T400X6S, T401X1A, T401X1D, T401X1S, T401X3A, T401X3D, T401X3S, T401X4A, T401X4D, T401X4S, T401X5A, T401X5D, T401X5S, T405X1A, T405X1D, T405X1S, T405X3A, T405X3D, T405X3S, T405X4A, T405X4D, T405X4S, T405X5A, T405X5D, T405X5S, T405X6A, T405X6D, T405X6S, T407X1A, T407X1D, T407X1S, T407X3A, T407X3D, T407X3S, T407X4A, T407X4D, T407X4S, T407X5A, T407X5D, T407X5S, T407X6A, T407X6D, T407X6S, T408X1A, T408X1D, T408X1S, T408X3A, T408X3D, T408X3S, T408X4A, T408X4D, T408X4S, T408X5A, T408X5D, T408X5S, T40901A, T40901D, T40901S, T40903A, T40903D, T40903S, T40904A, T40904D, T40904S, T40905A, T40905D, T40905S, T40906A, T40906D, T40906S, T40991A, T40991D, T40991S, T40993A, T40993D, T40993S, T40994A, T40994D, T40994S, T40995A, T40995D, T40995S, T40996A, T40996D, T40996S | APCD maternal medical claims |
| Alcohol use/abuse^f^ | 2910, 2911, 2912, 2913, 2914, 2915, 2918, 2919, 30300, 30301, 30302, 30303, 30390, 30391, 30392, 30393, 30500, 30501, 30502, 30503, 3575, 4255, 53530, 53531, 5710, 5711, 5712, 5713, 76071, 9800 | F1010, F10120, F10121, F10129, F1014, F10150, F10151, F10159, F10180, F10181, F10182, F10188, F1019, F1020, F1021, F10220, F10221, F10229, F10230, F10231, F10232, F10239, F1024, F10250, F10251, F10259, F1026, F1027, F10280, F10281, F10282, F10288, F1029, F10920, F10921, F10929, F1094, F10950, F10951, F10959, F1096, F1097, F10980, F10981, F10982, F10988, F1099, G621, I426, K2920, K2921, K700, K7010, K7011, K702, K7030, K7031, K7040, K709, O99310,^g^ O99311,^g^ O99312,^g^ O99313,^g^ O99314,^g^ O99315,^g^ P043, Q860 | Based on both reported history of alcohol intake extracted from BCR and medical claims of alcohol abuse extracted from APCD |
| Smoking^f^ | 3051, V1582 | F17, Z87891, O9933^g^ | Based on both reported history of smoking extracted from BCR, and medical claims of nicotine dependence extracted from APCD |
| Diabetes^h,i^ |  |  | Based on both reported history of diabetes extracted from BCR, and medical claims of diabetes extracted from APCD |
| 1. Diabetes mellitus without complications | 2500x, 2501x, 2502x, 2503x | E08, E09, E100x, E101x, E106xx, E108, E109, E110x, E111x, E116, E118, E119, E120x, E121x, E126x, E128, E129, E130x, E131x, E136xx, E138, E139, E140x, E141x, E146x, E148, E149 |  |
| 1. Diabetes mellitus with complications | 2504x, 2505x, 2506x, 2507x, 2508x, 2509x | E102x, E103xxx, E104x, E105x, E107, E112, E113, E114, E115, E117, E122, E123, E124, E125, E127, E132, E133, E134, E135, E137, E142, E143, E144, E145, E147, Z79.2 |  |
| 1. Gestational diabetes mellitus with or without pre-existing conditions | 648.8x | O24.0,^g^ O24.1,^g^ O24.3,^g^ O24.4,^g^ O24.8,^g^ O24.9 ^g^ |  |
| Placental pathologies or hemorrhage during pregnancy^h,i^ |  |  | Based on both reported history of placental pathologies extracted from BCR, and medical claims of placental pathologies extracted from APCD |
| 1. Placenta previa, complete or partial | 641.0x, 641.1x | O44.0,^g^ O44.1, ^g^ O44.2, ^g^ O44.3 ^g^ |  |
| 1. Placental abruption | 641.2 | O45^g^ |  |
| 1. Hemorrhage in pregnancy | 640.83, 640.93, | O208,^g^ O209,^g^ O46^g^ |  |
| Previous cesarean birth^h^ | 654.2 | O34.21^g^ | APCD maternal medical claims |
| Chronic hypertension or pregnancy induced hypertension^h^ | 6420, 6421, 6422, 6427, 401-405 | O10,^g^ O11,^g^ I10, O16^g^ | Based on both reported history of pregnancy induced hypertension extracted from BCR, and medical claims of pregnancy induced hypertension n extracted from APCD |
| Preeclampsia with or without eclampsia^h^ |  |  | Based on both reported history of preeclampsia/eclampsia extracted from BCR, and medical claims of these diagnoses extracted from APCD |
| 1. Preeclampsia with severe features including eclampsia | 642.5x, 642.6x | O14.1,^g^ O14.2,^g^ O15^g^ |  |
| 1. Preeclampsia without severe features or gestational hypertension | 642.3x, 642.4x, 642.7x | O13,^g^ O14.0,^g^ O14.9^g^ |  |
| Acute or chronic renal disease or pregnancy related renal disease |  |  | APCD medical claims |
| 1. Acute renal disease | 584.5, 584.6, 584.7, 584.8, 584.9, 669.3x | N17.x, O90.4^g^ |  |
| 1. Chronic renal disease | 581.x-583.x, 585.x, 587.x, 588.x, 646.2x | O26.83,^g^ I12, I13, N03- N05, N07, N08, N11.1, N11.8, N11.9, N18, N25.0, N25.1, N25.81, N25.89, N25.9, N26.9 |  |
| 1. Pregnancy related renal disease | 646.21, 646.22 | O26831,^g^ O26832,^g^ O26833^g^ |  |
| Cardiac disease^h^ | 745.0x-747.4x, 648.5x; 412.x-414.x; 428.22, 428.23, 428.32, 428.33, 428.42, 428.43, 394.x-397.x, 424.x | I05-I09, I11-I13, I15, I16, I20, I25, I27.8, I30- I41, I44-I49, I50.22, I50.23, I50.32, I50.33, I50.42, I50.43, I50.812, I50.813 O99.41,^g^ O99.42,^g^ Q20-Q24 | APCD maternal medical claims |
| Chorioamnionitis or other maternal infections excluding HIV^h,i^ |  |  | APCD maternal medical claims |
| 1. Chorioamnionitis or maternal intrauterine infection or maternal care for suspected damage to fetus secondary to viral disease in mom^h,i^ | 762.7 | O4110,^g^ O4112^g^ |  |
| 1. [Infective and parasitic](https://en.wikipedia.org/w/index.php?title=Infective_and_parasitic&action=edit&redlink=1) conditions in the mother (excluding HIV) ^h,i^ | 647.0-6 | O980-6,^g^ O98.8,^g^ O98.9^g^ |  |
| Sepsis or shock^i^ |  |  | APCD maternal medical claims |
| 1. Sepsis | 038.xx, 449, 785.52, 995.91, 995.92, 998.02, 670.2x | A32.7, A40.x, A41.x, I76, O85,^g^ O86.04,^g^ R65.20, R65.21, T81.12XA, T81.44XA |  |
| 1. Shock | 669.1x, 785.50, 785.51, 785.59, 995.0, 998.0, 998.00, 998.01, 998.09 | O75.1,^7^ R57.x, T78.2XXA, T81.10XA, T81.11XA, T81.19XA, T88.6XXA |  |
| Gastrointestinal disease^h^ | 520-579 | K (entire block), O99.6,^g^ O26.6^g^ | APCD maternal medical claims |
| Pulmonary pathologies^h^ |  |  | APCD maternal medical claims |
| 1. Asthma, acute or moderate/severe | 493.x | O99.5,^g^ J45.21, J45.22, J45.31, J45.32, J45.4, J45.5, J45.901, J45.902 |  |
| 1. Pulmonary hypertension | 416.0x, 416.8x, 416.9x | I27.0, I27.2 |  |
| Hematological pathologies^h^ |  |  | APCD maternal medical claims |
| 1. Bleeding disorder | 2824, 2826 | D66, D67, D68, D69 |  |
| 1. Blood loss anemia | 280, 281, 283, 284, 285, 286, 287, 288, 289, 7900, 7901, 79001, 79009, V123, V582 | D50, D55, D56, D58, D59, D571, D572, D573, D5740, D5780, D5720, O9901,^g^ O9902^g^ |  |
| Other maternal diagnoses^j^ |  |  | APCD maternal medical claims |
| 1. HIV | 042, 043x, 044x,V08 | B20, O98.7 |  |
| 1. Bariatric surgery | 43-44 | O99.84^g^ |  |
| 1. Thyrotoxicosis | 242.9 | E05 |  |
| 1. Cancer | 140.x-172.x, 174.x, 175.x, 179.x-195.x | C00, C01, C02, C03, C04, C05, C06, C07, C08, C09, C10, C12, C13, C14, C15, C16, C17, C18, C19, C20, C21, C22, C23, C25, C26, C30, C31, C32, C34, C35, C36, C37, C38, C39, C40, C41, C42, C43, C44, C50, C51, C52, C53, C54, C55, C56, C57, C58, C64, C65, C66, C67, C68, C69, C70, C71, C72, C73, C74, C75, C76, C77, C78, C79, C80, C81, C82, C83, C84, C85, C86, C87, C88, C89, C90, C91, C92, C93, C94, C95, C96 |  |
| *Maternal painful conditions* |  |  |  |
| Injuries^k^ |  |  | APCD maternal medical claims |
| 1. Fractures | 800, 801, 802, 803, 804, 805, 806, 807, 808, 809, 810, 811, 812, 813, 814, 815, 816, 817, 818, 819, 820, 821, 822, 823, 824, 825, 826, 827, 828, 829, 9050, 9051, 9052, 9053, 9054, 9055, V540, V5410, V5411, V5412, V5413, V5414, V5415, V5416, V5417, V5420, V5421, V5422, V5423 V5424, V5425, V5426, V5427, V5429 | S02, S07, S12, S22, S32, S42, S52, S62, S72, S82, S92, T02, T08, T10, T12, T14.2 |  |
| 1. Dislocations or sprains/strains | 830, 831, 832, 833, 834, 835, 836, 837, 838, 839, 840, 841, 842, 843, 844, 845, 846, 847, 848, 9057 | S03, S13, S23, S33, S43, S53, S63, S73, S83, S93, T03, T09.2, T11.2, T13.2, T14.3 |  |
| 1. Intracranial injury | 850, 851, 852, 853, 854 | S06, T06.0 |  |
| 1. Internal injury of chest, abdomen, and pelvis | 860, 861, 862, 863, 864, 865, 866, 867, 868, 869 | S26, S27, S36, S37, S39.6, T06.5 |  |
| 1. Open wounds | 870, 871, 872, 873, 874, 875, 876, 877, 878, 879, 880, 881, 882, 883, 884, 885, 886, 887, 888, 889, 890, 891, 892, 893, 894, 895, 896, 897 | S01, S05.2-S05.7, S08, S09.2, S11, S18, S21, S28.1, S31, S38.2, S38.3, S41, S48, S51, S58, S61, S68, S71, S78, S81, S88, S91, S98, T01, T05, T09.1, T09.6, T11.1, T11.6, T13.1, T13.6, T14.1, T14.7 |  |
| 1. Injury to blood vessels | 900, 901, 902, 903, 904 | S09.0, S15, S25, S35, S45, S55, S65, S75, S85, S95, T06.3, T11.4, T13.4, T14.5 |  |
| 1. Late effects of injury | 905, 906, 907, 908, 909 | T90, T91, T92, T93, T94, T95, T96, T97, T98 |  |
| 1. Crushing injury | 925, 926, 927, 928, 929 | S17, S28.0, S38.0, S38.1, S47, S57, S67, S77, S87, S97, T04 |  |
| 1. Effects of foreign body entering through orifice | 930, 931, 932, 933, 934, 935, 936, 937, 938, 939 | T15, T16, T17, T18, T19 |  |
| 1. Burns | 940, 941, 942, 943, 944, 945, 946, 947, 948, 949 | T20, T21, T22, T23, T24, T25, T26, T27, T28, T29, T30, T31, T32 |  |
| 1. Injury to nerve and spinal cord | 950, 951, 952, 953, 954, 955, 956, 957 | S04, S14, S24, S34, S44, S54, S64, S74, S84, S94, T06.1, T06.2, T09.3, T09.4, T11.3, T13.3, T14.4 |  |
| 1. Certain traumatic complications | 958 | T79 |  |
| 1. Injury, other and unspecified | 959 | S05, S09.7, S09.8, S09.9, S19, S29.7, S29.8, S29.9, S39.7, S39.8, S39.9, S49.7, S49.8, S49.9, S59.7, S59.8, S59.9, S69.7, S69.8, S69.9, S79.7, S79.8, S79.9, S89.7, S89.8, S89.9, S99.7, S99.8, S99.9, T06.8, T07, T09.8, T09.9, T11.8, T11.9, T13.8, T13.9, T14.8, T14.9 |  |
| 1. Transport accidents | E800-E879 | V01-V99 |  |
| 1. Falls | E880-E888 | W00-W19 |  |
| 1. Other and unspecified effects of external causes | 990, 991, 992, 993, 994, 995 | T33, T34, T35, T66, T67, T68, T69, T70, T71, T72, T73, T74, T75, T76, T77, T78 |  |
| 1. Complications of surgical and medical cause not elsewhere classified | 996, 997, 998, 999 | T80, T81, T82, T83, T84, T85, T86, T87, T88 |  |
| Arthropathies and connective tissue and autoimmune disorders | 710, 711, 712, 713, 714, 715, 716, 717, 718, 719, 274, 279.49 | M00, M01, M02, M03, M04, M05, M06, M07, M08, M09, M10, M11, M12, M13, M14, M15, M16, M17, M18, M19, M20, M21, M22, M23, M24, M26, M27, M28, M29, M30, M31, M32, M33, M34, M35, M36, M37, M38, M39, M40, M41, M42, M43, M44, M45, M46, M47, M48, M49, M50, M51, M52, M53, M55, M56, M57, M58, M59, M60, M61, M62, M63, M64, M65, M66, M67, M68, M69, M70, M71, M72, M73, M74, M75, M76, M77, M78, M79, M80, M81, M82, M83, M84, M85, M86, M87, M88, M89, M90, M91, M92, M93, M94, M95, M96, M97, M98, M99, M1A, D89.89 | APCD maternal medical claims |
| Headache/Migraine | 346xx, 339, 7840 | G43, G44, R51 | APCD maternal medical claims |
| Neuropathies or neuromuscular disease | 337.0, 350-359, 723.4, 727.2 | G40, G56, G57, G58, G59, G60, G61, G62, G63, G65, G70, O99.35^7^ | APCD maternal medical claims |
| *Maternal painful symptoms^l^* |  |  | APCD maternal medical claims |
| 1. Abdominal pain | 789.0, 789.1, 789.2, 789.3, 789.4, 789.5, 789.6, 789.9 | R10.0-6, R10.9 |  |
| 1. Central pain condition or pain not other classified | 338.0, 338.9 | R52, G89 |  |
| 1. Dental pain | 525.8, 525.9, 780.96 | K08.9, K08.89, K04.0 |  |
| 1. Chest pain | 786.50, 786.51, 786.52, 786.59 | R07.1, R07.2, R07.3, R07.9 |  |
| 1. Back and neck pain | 723.1, 724.2, 724.3, 724.4, 724.5, 847.0, 847.1, 847.2 | M54 |  |
| 1. Joint pain | 719.40-719.49 | M25 |  |
| 1. Kidney calculus pain | 592.0, 788.0 | N20.0, N23 |  |
| *Any maternal procedures^l^* | ICD-9 procedure codes with the first three or (more) digits as | ICD-10 procedure codes with the first three or (more) digits as | APCP maternal medical claims files |
|  | 001, 003, 004, 005, 006, 007, 008, 009, 010, 011, 012, 013, 014, 015, 016, 020, 021, 022, 023, 024, 029, 030, 031, 032, 033, 034, 035, 036, 037, 038, 039, 040, 041, 042, 043, 044, 046, 047, 048, 049, 050, 051, 052, 053, 058, 059, 060, 061, 062, 063, 064, 065, 066, 067, 068, 069, 070, 071, 072, 073, 074, 075, 076, 077, 078, 079, 080, 081, 082, 083, 084, 085, 086, 087, 088, 089, 090, 091, 092, 093, 094, 095, 096, 097, 098, 099, 100, 101, 102, 103, 104, 105, 106, 109, 110, 111, 112, 113, 114, 115, 116, 117, 119, 120, 121, 122, 123, 124, 125, 126, 127, 128, 129, 130, 131, 132, 133, 134, 135, 136, 137, 138, 139, 140, 141, 142, 143, 144, 145, 146, 147, 148, 149, 150, 151, 152, 153, 154, 155, 156, 157, 159, 160, 161, 162, 163, 164, 165, 166, 167, 168, 169, 171, 172, 173, 174, 175, 176, 177, 178, 180, 181, 182, 183, 184, 185, 186, 187, 189, 190, 191, 192, 193, 194, 195, 196, 199, 200, 201, 202, 203, 204, 205, 206, 207, 208, 209, 210, 211, 212, 213, 214, 215, 216, 217, 218, 219, 220, 221, 222, 223, 224, 225, 226, 227, 229, 230, 231, 232, 233, 234, 235, 236, 237, 240, 241, 242, 243, 244, 245, 246, 247, 248, 249, 250, 251, 252, 253, 254, 255, 259, 260, 261, 262, 263, 264, 269, 270, 271, 272, 273, 274, 275, 276, 277, 279, 280, 281, 282, 283, 284, 285, 286, 287, 289, 290, 291, 292, 293, 294,  295, 299, 300, 301, 302, 303, 304, 310, 311, 312, 313, 314, 315, 316, 317, 319, 320, 321, 322, 323, 324, 325, 326, 329, 330, 331, 332, 333, 334, 335, 336, 337, 339, 340, 341, 342, 343, 344, 345, 346, 347, 348, 349, 350, 351, 352, 353, 354, 355, 356, 357, 358, 359, 360, 361, 362, 363, 370, 371, 372, 373, 374, 375, 376, 377, 378, 379, 380, 381, 382, 383, 384, 385, 386, 387, 388, 389, 390, 391, 392, 393, 394, 395, 396, 397, 398, 399, 400, 401, 402, 403, 404, 405, 406, 409, 410, 411, 412, 413, 414, 415, 419, 420, 421, 422, 423, 424, 425, 426, 427, 428, 429, 430, 431, 433, 434, 435, 436, 437, 438, 439, 440, 441, 442, 443, 444, 445, 446, 449, 450, 451, 452, 453, 454, 455, 456, 457, 458, 459, 460, 461, 462, 463, 464, 465, 466, 467, 468, 469, 470, 471, 472, 479, 480, 481, 482, 483, 484, 485, 486, 487, 488, 489, 490, 491, 492, 493, 494, 495, 496, 497, 499, 500, 501, 502, 503, 504, 505, 506, 509, 510, 511, 512, 513, 514, 515, 516, 517, 518, 519, 520, 521, 522, 523, 524, 525, 526, 527, 528, 529, 530, 531, 532, 533, 534, 535, 536, 539, 540, 541, 542, 543, 544, 545, 546, 547, 549, 550, 551, 552, 553, 554, 555, 556, 557, 558, 559, 560, 561, 562, 563, 564, 565, 566, 567, 568, 569, 570, 571, 572, 573, 574, 575, 576, 577, 578, 579, 580, 581, 582, 583, 584, 585, 586, 589, 590, 591, 592, 593, 594, 595, 596, 597, 598, 599, 610, 611, 612, 619, 645, 650, 651, 652, 653, 654, 655, 656, 657, 658, 659, 660, 661, 662, 663, 664, 665, 666, 667, 668, 669, 670, 671, 672, 673, 674, 675, 676, 680, 681, 682, 683, 684, 685, 686, 687, 688, 689, 690, 691, 692, 693, 694, 695, 696, 697, 699, 700, 701, 702, 703, 704, 705, 706, 707, 709, 710, 711, 712, 713, 714, 715, 716, 717, 718, 751, 752, 753, 760, 761, 762, 763, 764, 765, 766, 767, 769, 770, 771, 772, 773, 774, 775, 776, 777, 778, 779, 780, 781, 782, 783, 784, 785, 786, 787, 788, 789, 790, 791, 792, 793, 794, 795, 796, 797, 798, 799, 800, 801, 802, 803, 804, 805, 806, 807, 808, 809, 810, 811, 812, 813, 814, 815, 816, 817, 818, 819, 820, 821, 822, 823, 824, 825, 826, 827, 828, 829, 830, 831, 832, 833, 834, 835, 836, 837, 838, 839, 840, 841, 842, 843, 844, 845, 846, 847, 848, 849, 850, 851, 852, 853, 854, 855, 856, 857, 858, 859, 860, 861, 862, 863, 864, 865, 866, 867, 868, 869, 870, 871, 872, 873, 874, 875, 876, 877, 878, 879, 880, 881, 882, 883, 884, 885, 886, 892, 894, 922, 923, 924, 930, 931, 932, 933, 934, 935, 936, 961, 962, 963, 964, 965, 966, 967, 970, 971, 972, 974, 975, 976, 977, 978, 980, 981, 982, 985, 990, 991, 992, 996, 9999 | **Drainage procedure codes with the first three or (more) digits as:** 009, 019, 039, 049, 059, 069, 079, 089, 099, 0B9, 0C9, 0D9, 0F9, 0G9, 0H9, 0J9, 0K9, 0L9, 0M9, 0N9, 0P9, 0Q9, 0R9, 0S9, 0T9, 0U9, 0W9, 0X9, 0Y9, 0V9630Z, 0V963ZZ, 0V9730Z, 0V973ZZ |  |
|  |  | **Extraction procedure codes with the first three or (more) digits as:** 0BD, 0CD, 0DD, 0FD, 0HD, 0JD, 0KD, 0LD, 0MD, 0ND, 0PD, 0QD, 0UD, 00D, 01D, 05D, 06D, 07D, 08D, 09D |  |
|  |  | **Insertion procedure codes with the first three or (more) digits as:** 00H, 01H, 02H, 03H, 04H, 05H, 06H, 07H, 08H, 09H, 10H, 0BH, 0CH, 0DH, 0FH, 0GH, 0HH, 0JH, 0KH, 0LH, 0NH, 0PH, 0QH, 0RH, 0SH, 0TH, 0UH, 0WH, 0XH, 0YH, 0VH833Z, 0VH83YZ, 0VH87YZ, 0VH883Z, 0VH88YZ, X0H, X2H, XNH, XRH |  |
|  |  | **Excision procedure codes with the first three or (more) digits as:** 00B, 01B, 02B, 03B, 04B, 05B, 06B, 07B, 08B, 09B, 0BB, 0CB, 0DB, 0FB, 0GB, 0HB, 0JB, 0KB, 0LB, 0MB, 0NB, 0PB, 0QB, 0RB, 0SB, 0TB, 0UB, 0WB, 0XB, 0YB, 0VB6, 0VB7 |  |
|  |  | **Inspection procedure codes with the first three or (more) digits as:** 00J, 01J, 02J, 03J, 04J, 05J, 06J, 07J, 08J, 09J, 10J, 0BJ, 0CJ, 0DJ, 0FJ, 0GJ, 0HJ, 0JJ, 0KJ, 0LJ, 0MJ, 0NJ, 0PJ, 0QJ, 0RJ, 0SJ, 0TJ, 0UJ, 0WJ, 0XJ, 0YJ, 0VJ8 |  |
|  |  | **Bypass procedure codes with the first three or (more) digits as:** 0016, 0210, 0211, 0212, 0213, 0216, 0217, 0312, 0313, 0314, 0315, 0316, 0317, 0318, 0319, 0410, 0413, 0414, 0610, 0611, 0618, 091E, 041E, 001U, 021K, 021L, 021P, 021Q, 021R, 021V, 021W, 021X, 031A, 031B, 031C, 031G, 031H, 031J, 031K, 031L, 031M, 031N, 031S, 031T, 041C, 041D, 041E, 041F, 041H, 041J, 041K, 041L, 041M, 041N, 041P, 041Q, 041R, 041S, 041T, 041U, 041V, 041W, 051, 061, 071, 081, 091, 0B1, 0D1, 0F1, 0T1, 0U1, 0W1, X2K |  |
|  |  | **Dilation procedure codes with the first three or (more) digits as:** 007, 027, 037, 047, 057, 067, 087, 097, 0B7, 0C7, 0D7, 0F7, 0T7, 0U7, X27 |  |
|  |  | **Destruction procedure codes with the first three or (more) digits as:** 005, 015, 025, 035, 045, 055, 065, 075, 085, 095, 0B5, 0C5, 0D5, 0F5, 0G5, 0H5, 0J5, 0K5, 0L5, 0M5, 0N5, 0P5, 0Q5, 0R5, 0S5, 0T5, 0U5, X05, XV5 |  |
|  |  | **Division procedure codes with the first three or (more) digits as:** 008, 018, 028, 098, 0D8, 0F8, 0G8, 0H8, 0J8, 0K8, 0L8, 0M8, 0N8, 0P8, 0Q8, 0T8, 0U8, X28, 0W8 |  |
|  |  | **Extirpation procedure codes with the first three or (more) digits as:** 00C, 01C, 01D, 02C, 03C, 04C, 05C, 06C, 07C, 08C, 09C, 0BC, 0CC, 0DC, 0FC, 0GC, 0HC 0JC, 0KC, 0LC, 0MC, 0NC, 0PC, 0QC, 0RC, 0SC, 0TC, 0UC, 0WC, X2C, 0VC63ZZ, 0VC73ZZ |  |
|  |  | **Fragmentation procedure codes with the first three or (more) digits as:** 00F, 02F, 03F, 04F, 05F, 06F, 08F, 0BF, 0CF, 0DF, 0FF, 0TF, 0UF, 0WF |  |
|  |  | **Removal procedure codes with the first three or (more) digits as:** 00P, 01P, 02P, 03P, 04P, 05P, 06P, 07P, 08P, 09P, 0BP, 0CP, 0DP, 0FP, 0GP, 0HP, 0JP, 0KP, 0LP, 0MP, 0NP, 0PP, 0QP, 0RP, 0SP, 0TP, 0UP, 0WP, 0VP8, 0XP, 0YP, 2W5, 2Y5 |  |
|  |  | **Release procedure codes with the first three or (more) digits as:** 00N, 01N, 02N, 03N, 04N, 05N, 06N, 07N, 08N, 09N, 0BN, 0CN, 0DN, 0FN, 0GN, 0HN, 0JN, 0KN, 0LN, 0MN, 0NN, 0PN, 0QN, 0RN, 0SN, 0TN, 0UN |  |
|  |  | **Transfer procedure codes with the first three or (more) digits as:** 00X, 01X, 08X, 0CX, 0DX, 0HX, 0JX, 0KX, 0LX, 0MX, 0XX |  |
|  |  | **Restriction procedure codes with the first three or (more) digits as:** 02V, 03V, 04V, 05V, 06V, 07V, 08V, 0BV, 0CV, 0DV, 0FV, 0TV, 0UV, X2V |  |
|  |  | **Reposition procedure codes with the first three or (more) digits as:** 00S, 01S, 02S, 03S, 04S, 05S, 06S, 07S, 08S, 09S, 0BS, 0CS, 0DS, 0FS, 0GS, 0HS, 0KS, 0LS, 0MS, 0NS, 0PS, 0QS, 0RS, 0SS, 0TS, 0US, XNS |  |
|  |  | **Replacement procedure codes with the first three or (more) digits as:** 00R, 01R, 02R, 03R, 04R, 05R, 06R, 08R, 09R, 0BR, 0CR, 0DR, 0FR, 0HR, 0JR, 0KR, 0LR, 0MR, 0NR, 0PR, 0QR, 0RR, 0SR, 0TR, 0XR, X2R, XHR, XNR, XRR |  |
|  |  | **Resection procedure codes with the first three or (more) digits as**: 00T, 02T, 07T, 08T, 09T, 0BT, 0CT, 0DT, 0FT, 0GT, 0HT, 0KT, 0LT, 0MT, 0NT, 0PT, 0QT, 0RT, 0ST, 0TT, 0UT, 0VT6, 0VT7 |  |
|  |  | **Supplement procedure codes with the first three or (more) digits as**: 00U, 01U, 02U, 03U, 04U, 05U, 06U, 07U, 08U, 09U, 0BU, 0CU, 0DU, 0FU, 0HU, 0JU, 0KU, 0LU, 0MU, 0NU, 0PU, 0QU, 0RU, 0SU, 0TU, 0UU, 0VU6, 0WU, 0XU, 0YU, X2U, XKU, XNU |  |
|  |  | **Transplantation procedure codes with the first three or (more) digits as**: 02Y, 07Y, 0BY, 0DY, 0FY, 0TY, 0UY, 0WY, 0XY, 0DY |  |
|  |  | **Occlusion procedure codes with the first three or (more) digits as**: 02L, 03L, 04L, 05L, 06L, 07L, 08L, 0BL, 0CL, 0DL, 0FL, 0TL, 0UL, 02L |  |
|  |  | **Reattachment procedure codes with the first three or (more) digits as:** 08M, 09M, 0BM, 0CM, 0DM, 0FM, 0GM, 0HM, 0KM, 0LM, 0MM, 0TM, 0UM, 0VM6, 0VM7, 0WM, 0XM, 0YM |  |
|  |  | **Alteration procedure codes with the first three or (more) digits as:** 080, 090, 0C0, 0H0, 0J0, 0W0, 0X0, 0Y0 |  |
|  |  | **Photon radiosurgery procedure codes with the first three or (more) digits as:** D02, D72, D82, D92, DB2, DD2, DF2, DG2, DM2, DT2, DU2, DW2 |  |
|  |  | **Beam radiation or other radiation codes with the first three or (more) digits as:** D00, D0Y, D70, D7Y, D80, D8Y, D90, D9Y, DB0, DBY, DD0, DDY, DF0, DFY, DG0, DGY, DH0, DYI, DM0, DMY, DP0, DPY, DT0, DTY, DU0, DUY, DW0, DWY |  |
|  |  | **Robotic assisted procedure or acupuncture:** 8E09, 8E0H, 8E0W, 8E0X, 8E0Y |  |
|  |  | **Chiropractic codes with the first three or (more) digits as:** 9WB0, 9WB1, 9WB2, 9WB3, 9WB4, 9WB5, 9WB6, 9WB7, 9WB8, 9WB9 |  |
|  |  | **Laser procedure codes with the first three or (more) digits as:** D0Y, DBY, DDY, DFY, DGY, DMY |  |
|  |  | **Detachment procedure codes with the first three or (more) digits as:** 0X6, 0Y6 |  |
|  |  | **Alteration** **procedure codes with the first three or (more) digits as:** 080, 090, 0C0, 0H0, 0J0, 0W0, 0X0, 0Y0 |  |
|  |  | **Brachytherapy procedure codes with the first three or (more) digits as:** D01, D71, D81, D91, DB1, DD1, DF1, DG1, DM1, DT1, DU1, DW1 |  |
|  |  | **Fluoroscopy procedure codes with the first three or (more) digits as:** B21, B31, B41, B51, B91, BB1, BD1, BF1, BN1, BP1, BQ1, BR1, BT1, BU1, BW1 |  |
|  |  | **Fusion procedure codes with the first three or (more) digits as:** 10H, 0RG, 0SG, XRG |  |
|  |  | **Control bleeding procedure codes with the first three or (more) digits as:** 0W3, 0X3, 0Y3, 093 |  |
|  |  | **Change procedure codes with the first three or (more) digits as:** 002, 012, 072, 082, 092, 0B2, 0C2, 0D2, 0F2, 0G2, 0H2, 0J2, 0K2, 0L2, 0M2, 0N2, 0P2, 0Q2, 0R2, 0S2, 0T2, 0U2, 0W2, 0X26, 0X27, 0Y2 |  |
|  |  | **Change traction, splint, cast, brace, bandage, packing, pressure with intermittent device pressure or another device procedure codes with the first three or (more) digits as:** 2W00, 2W01, 2W02, 2W03, 2W04, 2W05, 2W06, 2W07, 2W08, 2W09, 2W0A, 2W0B, 2W0C, 2W0D, 2W0E, 2W0F, 2W0G, 2W0H, 2W0J, 2W0K, 2W0L, 2W0M, 2W0N, 2W0P, 2W0Q, 2W0R, 2W0S, 2W0T, 2W0U, 2W0V |  |
|  |  | **Mapping, measurement, monitoring, introduction, filtration, dressing, compression, immobilization, packing or change or removal of packing material, traction, wound management or, irrigation procedure codes with the first three or (more) digits as:** 00K, 02K, 4A0, 4A1, 4B0, 3E0, 3E1, F08, F0C, 2W1, 2W2, 2W3, 2W4, 2W6, 3E0, XD2, XW0, 2Y0, 2Y4, X2A, 3E0Q0KZ, XR2G021, XR2H021, 3C1ZX8Z |  |
|  |  | **Other procedure codes with the first three or (more) digits as:** 024, 6AB, 5A0, 5A1, 5A2**,** F07, 8C0, CW7, 7W0, XW0, XW1, 8E0, X0Z0X18, X2A7358, X2H03R9, XDPH8K7, XF50X08, XF51X08, XF52X08, XK02303, XW23346, XW23376, XW24346, XW24376, XWHD7Q7, XXA536A, XY0VX83, XY0YX28, XY0YX37 |  |
|  |  | |  |
| ***Pharmacological agents*** | **Drug codes** | | **Source** |
| *Buprenorphine* | **NDC codes**: 54017613, 54017713, 54018813, 54018913, 93360021, 93360040, 93360121, 93360140, 93360221, 93360240, 93360321, 93360340, 93537856, 93537956, 93572056, 93572156, 228315303, 228315403, 228315473, 228315503, 228315573, 228315603, 378092393, 378092493, 406192303, 406192403, 490005100, 490005130, 490005160, 490005190, 12496120201, 12496120203, 12496120803, 12496121201, 12496121203, 12496127802, 12496128302, 12496130602, 12496131002, 16590066605, 16590066630, 16590066705, 16590066730, 35356055530, 35356055630, 35356060504, 35356060604, 35356060704, 42291017430, 42291017530, 42858035340, 42858049340, 42858058640, 42858075040, 42858083940, 43063018407, 43063018430, 43063066706, 43063075306, 49999039507, 49999039515, 49999039530, 49999063830, 49999063930, 50268014411, 50268014415, 50268014511, 50268014515, 50383028793, 50383029493, 50383092493, 50383093093, 52959030430, 52959074930, 53217013830, 53217024630, 54123011430, 54123090730, 54123091430, 54123092930, 54123095730, 54123098630, 54569549600, 54569573900, 54569573901, 54569573902, 54569632500, 54569632600, 54569639900, 54569640800, 54569657800, 54868570700, 54868570701, 54868570702, 54868570703, 54868570704, 54868575000, 55045378403, 55700014730, 55700018430, 55700030230, 55700030330, 55887031204, 55887031215, 58284010014, 59011075004, 59011075104, 59011075204, 59011075704, 59011075804, 59385001201, 59385001230, 59385001401, 59385001430, 59385001601, 59385001630, 59385002160, 59385002360, 59385002460, 59385002560, 59385002760, 60429058630, 60429058633, 60429058730, 60429058733, 62756045983, 62756046083, 63481016160, 63481020760, 63481034860, 63481051960, 63481068560, 63481082060, 63481095260, 63629403401, 63629403402, 63629403403, 63629409201, 63874108403, 63874108503, 63874117303, 63874117403, 65162041503, 65162041603, 66336001530, 68071138003, 68071151003, 68258299103, 68258299903, 68308020230, 68308020830, 228315567, 406192309, 406192409, 406800503, 406802003, 12496010001, 12496030001, 12496030002, 12496030005, 42858050103, 42858050203, 50090157100, 55700056804, 55700057904, 59385002260, 59385002601, 59385002660, 60846097003, 60846097103, 62175045232, 62175045832, 62756045964, 62756046064, 62756096964, 62756096983, 62756097064, 62756097083, 63629409202, 63629507401, 63629712501, 63629712502, 63629712503, 63629712504, 63629712505, 63629712506, 63629712507, 63629712601, 63629712602, 63629712603, 63629712604, 63629712605, 63629712606, 63629712607, 63629712608, 63629727001, 63629727002, 64725093003, 64725093004, 64725192403, 64725192404, 65162041509, 65162041609, 71335035301, 71335035302, 71335035303, 71335035304, 71335035305, 71335035306, 71335035307, 76519117000, 76519117001, 76519117002, 76519117003, 76519117004, 54309036, 87565041, 378963943, 403479118, 54569368100, 54569598800, 54868320900, 54868458300, 55175441601, 58016483301, 59911594401, 60505081301 | | APCD pharmacy claims/PDMD. NDCs were used to identify buprenorphine^a^ |
| *Benzodiazepines^m^* | 8-digit GPI | | APCD pharmacy claims. GPIs were used to identify drugs |
| 1. Alprazolam | 57100010 | |  |
| 1. Chlordiazepoxide | 57100020 | |  |
| 1. Clorazepate dipotassium | 57100030 | |  |
| 1. Diazepam | 57100040 | |  |
| 1. Diazepam (anticonvulsant) | 72100030 | |  |
| 1. Halazepam | 57100050 | |  |
| 1. Lorazepam | 57100060 | |  |
| 1. Oxazepam | 57100070 | |  |
| 1. Alprazolam dietary management product | 57999002 | |  |
| 1. Midazolam/Midazolam HCL/Midazolam sodium chloride | 60201025 | |  |
| 1. Midazolam (anticonvulsant) | 72100060 | |  |
| 1. Remimazolam Besylate | 60201029 | |  |
| 1. Estazolam | 60201005 | |  |
| 1. Flurazepam HCL | 60201010 | |  |
| 1. Temazepam | 60201030 | |  |
| 1. Triazolam | 60201040 | |  |
| *Selective serotonin reuptakn^n^ inhibitor (SSRI)* | 8-digit GPI | | APCD pharmacy claims. GPIs were used to identify drugs |
| 1. Vilazodone HCL | 58120088 | |  |
| 1. Vortioxetine HBR | 58120093 | |  |
| 1. Citalopram hydrobromide | 58160020 | |  |
| 1. Escitalopram oxalate | 58160034 | |  |
| 1. Fluoxetine HCL | 58160040 | |  |
| 1. Fluvoxamine Maleate | 58160045 | |  |
| 1. Paroxetine HCL/Paroxetine Mesylate | 58160060 | |  |
| 1. Sertraline HCL | 58160070 | |  |
| 1. Fluoxetine HCL-Dietary management product | 58998502 | |  |
| *Tricyclic antidepressant pharmacological agents other than SSR^q^* | 8-digit GPI | | APCD pharmacy claims. GPIs were used to identify drugs |
| 1. Tricyclics and Tetracyclics |  | |  |
| 1. Mirtazapine | 58030050 | |  |
| 1. Amitriptyline HCL | 58200010 | |  |
| 1. Clomipramine HCL | 58200025 | |  |
| 1. Desipramine HCL | 58200030 | |  |
| 1. Doxepin HCL | 58200040 | |  |
| 1. Imipramine HCL/Impramine Pamoate | 58200050 | |  |
| 1. Nortriptyline HCL | 58200060 | |  |
| 1. Protriptyline HCL | 58200070 | |  |
| 1. Trimipramine Maleate | 58200080 | |  |
| 1. Maprotiline HCL | 58300010 | |  |
| 1. Antidepressants in combination with other agents |  | |  |
| 1. Amitriptyline/Chlordiazepoxide/Perphenazine w/Amitriptyline/Chlordiazepoxide-Amitriptyline | 62990002 | |  |
| 1. Perphenazine-Amitriptyline | 62994002 | |  |
| 1. Newer Generation Antidepressant |  | |  |
| 1. Bupropion HCL/Bupropion Hydrobromide | 58300040 | |  |
| 1. Bupropion HCL-Dietary management product/Dextromethorphan Hydrobromide-Bupropion Hydrochloride | 58999002 | |  |
| 1. Antidepressants, others |  | |  |
| 1. Brexanolone | 58060015 | |  |
| 1. Zuranolone | 58060090 | |  |
| 1. Esketamine HCL | 58110020 | |  |
| 1. Nefazodone HCL | 58120050 | |  |
| 1. Trazodone HCL | 58120080 | |  |
| 1. Trazodone HCL-Dietary management product | 58998002 | |  |
| 1. Monoamine oxidase inhibitors |  | |  |
| 1. Isocarboxazid | 58100010 | |  |
| 1. Phenelzine Sulfate | 58100020 | |  |
| 1. Selegiline | 58100027 | |  |
| 1. Tranylcypromine Sulfate | 58100030 | |  |
| 1. Serotonin Receptor Modulator |  | |  |
| 1. Fluoxetine HCL | 62206040 | |  |
| 1. Paroxetine Mesylate | 62226060 | |  |
| 1. Serotonin Norepinephrine Reuptake Inhibitors |  | |  |
| 1. Desvenlafaxine/Desvenlafaxime Fumarate | 58180020 | |  |
| 1. Duloxetine HCL | 58180025 | |  |
| 1. Levomilnacipran HCL | 58180050 | |  |
| 1. Venlafaxine Besylate/Venlafaxine HCL | 58180090 | |  |
| 1. Milnacipran HCL | 62504050 | |  |
| *Gamma-aminobutyric acid pharmacological agents^r^* | 8-digit GPI | | APCD pharmacy claims. GPIs were used to identify drugs |
| 1. Gabapentin | 62540030 | |  |
| 1. Pregabalin (once daily) | 62540060 | |  |
| 1. Gabapentin and Lidocaine-Menthol | 62549903 | |  |
| 1. Gabapentin Enacarbil | 62560030 | |  |
| 1. Pregabalin | 72600057 | |  |
| 1. Gabapentin-Dietary management product | 72996002 | |  |
| *Amphetamine pharmacological agents*^s^ | 8-digit GPI | | APCD pharmacy claims. GPIs were used to identify drugs |
| 1. Amphetamine/Amphetamine Sulfate | 61100010 | |  |
| 1. Dextroamphetamine | 61100020 | |  |
| 1. Lisdexamfetamine Dimesylate | 61100025 | |  |
| 1. Methamphetamine HCL | 61100030 | |  |
| 1. Amphetamine-Dextroamphetamine | 61109902 | |  |
| *Antipsychotic pharmacological agents^t^* | 8-digit GPI | | APCD pharmacy claims. GPIs were used to identify drugs |
| 1. Iloperidone | 59070035 | |  |
| 1. Paliperidone/Paliperidone Palmitate | 59070050 | |  |
| 1. Risperidone/Risperidone Microspheres | 59070070 | |  |
| 1. Haloperidol/Haloperidol Lactate | 59100010 | |  |
| 1. Clozapine | 59152020 | |  |
| 1. Quetiapine Fumarate | 59153070 | |  |
| 1. Loxapine/Loxapine succinate | 59154020 | |  |
| 1. Asenapine/Asenapine Maleate | 59155015 | |  |
| 1. Olanzapine/Olanzapine Pamoate | 59157060 | |  |
| 1. Molindone HCL | 59160050 | |  |
| 1. Aripiprazole/Aripiprazole with sensor/Aripiprazole Lauroxil | 59250015 | |  |
| 1. Brexpiprazole | 59250020 | |  |
| 1. Thiothixene/Thiothixene HCL | 59300020 | |  |
| 1. Molindone HCL | 59400010 | |  |
| 1. Cariprazine HCL | 59400018 | |  |
| 1. Loxapine Succinate | 59400020 | |  |
| 1. Lumateperone Tosylate | 59400022 | |  |
| 1. Lurasidone HCL | 59400023 | |  |
| 1. Pimavanserin Tartrate | 59400028 | |  |
| 1. Ziprasidone HCL/Ziprasidone Mesylate | 59400085 | |  |
| 1. Pimozide | 62000030 | |  |
| 1. Olanzapine-Samidorphan L-Malate | 62994802 | |  |
| 1. Olanzapine-Fluoxetine HCL | 62995002 | |  |
| 1. Chlorpromazine/Chlorpromazine HCL | 59200015 | |  |
| 1. Fluphenazine HCL/Fluphenazine Decanoate | 59200025 | |  |
| 1. Mesoridazine Besylate | 59200030 | |  |
| 1. Perphenazine | 59200045 | |  |
| 1. Promazine HCL | 59200060 | |  |
| 1. Thioridazine HCL | 59200080 | |  |
| 1. Trifluoperazine HCL | 59200085 | |  |
| 1. Prochlorperazine/Prochlorp-erazine maleate/Prochlorperazine Edisylate | 59200055 | |  |
| 1. Lithium/Lithium Carbonate/Lithium Citrate | 59500010 | |  |
| *Barbiturate^u^* | 8-digit GPI | | APCD pharmacy claims. GPIs were used to identify drugs |
| 1. Amobarbital Sodium | 1. 60100010 | |  |
| 1. Butobarbital Sodium | 1. 60100025 | |  |
| 1. Mephobarbital | 1. 60100040 | |  |
| 1. Phenobarbital/Phenobarbital Sodium | 1. 60100040 | |  |
| 1. Pentobarbital Sodium | 1. 60100055 | |  |
| 1. Secobarbital Sodium | 1. 60100070 | |  |
| *Beta-Blockers^v^* | 2-digit GPI (the first 2 digit of GPI): 33 | | APCD pharmacy claims. GPIs were used to identify drugs |
| *Thyroid pharmacological agents^w^* | 2-digit GPI (the first 2 digit of GPI): 28 | | APCD pharmacy claims. GPIs were used to identify drugs |
| *Macrolides, Tetracyclines, and fluoroquinolones pharmacological agents^x^* | 2-digit GPI (the first 2 digit of GPI): 03, 04, 05 | | APCD pharmacy claims. GPIs were used to identify drugs |
|  |  | |  |
| **Domains of social determinant of health^y,z^** | **Description of variable** | | **Source** |
|  |  | | SDOH variables linked to the 5-digit zip code of maternal address at time of delivery |
| ***Economic stability*** |  | | SDOH variables linked to the 5-digit zip code of maternal address at time of delivery |
| ACS_MEDIAN_HH_INC_ZC | Median household income (dollars, inflation-adjusted to data file year), all adjusted to $2016 | |  |
| ACS_PCT_HH_FOOD_STMP_BLW_POV_ZC | Percentage of households receiving food stamps/SNAP with income below the poverty level | |  |
| ACS_PCT_HU_NO_VEH_ZC | Percentage of housing units with no vehicle available | |  |
| ***Education access and quality*** |  | |  |
| ACS_PCT_LT_HS_ZC | Percentage of population with less than high school education (ages 25 and over) | |  |
| ACS_PCT_HS_GRADUATE_ZC | Percentage of population with only high school diploma (ages 25 and over) | |  |
| ***Heath care access and quality*** |  | |  |
| ACS_PCT_MEDICAID_ANY_ZC | Percentage of population with any Medicaid/means-tested public health insurance coverage | |  |
| ACS_PCT_UNINSURED_ZC | Percentage of population with no health insurance coverage | |  |
| POS_DIST_CLINIC_ZP | Distance in miles to the nearest health clinic (FQHC, RHC), calculated using population weighted ZIP centroids | |  |
| POS_DIST_ED_ZP | Distance in miles to the nearest emergency department, calculated using population weighted ZIP centroids | |  |
| ***Neighborhood and built environment*** |  | |  |
| ACS_PCT_HU_PLUMBING_ZC | Percentage of housing units lacking complete plumbing facilities | |  |
| ACS_PCT_HU_BUILT_1979_ZC | Percentage of housing units built before 1979 | |  |
| ERS_RUCA1_2010_ZP | Primary Rural-Urban Commuting Area Code 2010 | |  |
| ***Social and community context*** |  | |  |
| ACS_AVG_HH_SIZE_ZC | Average household size | |  |
| ACS_PCT_FOREIGN_BORN_ZC | Percentage of population that is foreign-born | |  |
| ACS_PCT_HH_LIMIT_ENGLISH_ZC | Percentage of limited English-speaking households | |  |
| ACS_PCT_HOUSEHOLDER_WHITE_ZC | Percentage of householders who are White alone | |  |
| ACS_PCT_HOUSEHOLDER_BLACK_ZC | Percentage of householders who are Black or African American alone | |  |

Abbreviations (by alphabetical order): APCD, All Payer Claims Database; BCR, birth certificate records; BMI, body mass index; GPI, generic product identifier; ICD-9-CM, International Classification of Diseases, 9^th^ Revision, Clinical Modification; ICD-9-CM, International Classification of Diseases, 10^th^ Revision, Clinical Modification; MME, morphine milligram equivalent; NDC, National Drug Code; PDMP, prescription drug monitoring program; PPV, positive predictive value; RUCA, Rural-Urban Commuting Area; SDOH, Social Determinant of Health Database; SSRI, Selective serotonin reuptake inhibitor; WIC, Special Supplemental Nutrition Program for Women, Infants, and Children

^a^Dowell D, Ragan KR, Jones CM, Baldwin GT, Chou R. CDC clinical practice guideline for prescribing opioids for pain - United States, 2022. MMWR Recomm Rep 2022; 71(3):1-95.

^b^Andrade SE, Scott PE, Davis RL, Li DK, Getahun D, Cheetham TG et al. Validity of health plan and birth certificate data for pregnancy research. Pharmacoepidemiol Drug Saf 2013; 22(1):7-15.

^c^Olsen IE, Groveman SA, Lawson ML, Clark RH, Zemel BS. New intrauterine growth curves based on United States data. Pediatrics 2010; 125(2):e214-24.

^d^Maalouf FI, Cooper WO, Stratton SM, Dudley JA, Ko J, Banerji A, Patrick SW. Positive predictive value of administrative data for neonatal abstinence syndrome. Pediatrics 2019; 143(1): e20174183.

^e^RUCA subdivision was the US Department of Agriculture summary on 2010 RUCA available at https://www.ers.usda.gov/data-products/rural-urban-commuting-area-codes/documentation.

^f^Substance Abuse and Mental Health Services Administration. Appendix E Mental Health and Substance Use Diagnosis. <https://www.samhsa.gov/data/sites/default/files/reports/rpt29396/2018-MHCLD/2018-MHCLD-AppE.pdf>

^g^ICD10 codes that start with “O” indicate diagnosis during pregnancy.

^h^Leonard SA, Kennedy CJ, Carmichael SL, Lyell DJ, Main EK. An expanded obstetric comorbidity scoring system for predicting severe maternal morbidity. Obstet Gynecol. 2020;136(3):440-449. doi: 10.1097/AOG.0000000000004022.

^i^Centers for Disease Control and Prevention. Severe maternal morbidity. <https://www.cdc.gov/maternal-infant-health/php/severe-maternal-morbidity/index.html>

^j^Rare diagnoses were grouped into a single category due to their low frequency of occurrence.

^k^Centers for Disease Control and Prevention. The ICD-IO Classifications of Injuries and External Causes. <https://www.cdc.gov/nchs/data/ice/ice95v1/c22.pdf>

^l^Codes were summarized per authors’ review of ICD-9-CM and ICD-10-CM on <https://hcup-us.ahrq.gov/toolssoftware/ccs/ccs.jsp> and <https://hcup-us.ahrq.gov/toolssoftware/ccsr/dxccsr.jsp>

^m^Sanlorenzo LA, Cooper WO, Dudley JA, Stratton S, Maalouf FI, Patrick SW. Increased severity of neonatal abstinence syndrome associated with concomitant antenatal opioid and benzodiazepine Exposure. Hosp Pediatr 2019; 9(8):569-75.

^n^Eke AC, Saccone G, Berghella V. Selective serotonin reuptake inhibitor (SSRI) use during pregnancy and risk of preterm birth: a systematic review and meta-analysis. BJOG 2016; 123(12):1900-7.

^q^The Agency for Healthcare Rsearch and Quality. Antidepressant treatment of depression during pregnancy and the postpartum period [updated 2014 Jul 8; cited Mar 28]. Available from: <https://effectivehealthcare.ahrq.gov/sites/default/files/related_files/depression-pregnancy-postpartum_executive.pdf>

^r^Patorno E, Hernandez-Diaz S, Huybrechts KF, Desai RJ, Cohen JM, Mogun H, et al. Gabapentin in pregnancy and the risk of adverse neonatal and maternal outcomes: A population-based cohort study nested in the US Medicaid Analytic eXtract dataset. PLoS Med 2020; 17(9):e1003322.

^s^Raffi ER, Nonacs R, Cohen LS. Safety of Psychotropic Medications During Pregnancy. Clin Perinatol 2019; 46(2):215-34.

The American College of Obstetrics and Gynecology. Committee Opinion No.479. Methamphetamine abuse in women of reproductive age. Obstet Gynecol 2011; 117(3):751-5.

^t^Betcher HK, Montiel C, Clark CT. Use of antipsychotic drugs during pregnancy. Curr Treat Options Psychiatry 2019; 6(1):17-31.

^u^Pandey SK, Dass D. Drugs of physical harm in pregnancy: Nature vs nurture-a silent battle. J Appl Pharm Sci 2012; 6:11-9.

^v^Fitton CA, Steiner MFC, Aucott L, Pell JP, Mackay DF, Fleming M, et al. In-utero exposure to antihypertensive medication and neonatal and child health outcomes: a systematic review. J Hypertens 2017; 35(11):2123-37.

^w^Derakhshan A, Peeters RP, Taylor PN, Bliddal S, Carty DM, Meems M, et al. Association of maternal thyroid function with birthweight: a systematic review and individual-participant data meta-analysis. Lancet Diabetes Endocrinol 2020; 8(6):501-10.

^x^Nguyen MH, Fornes R, Kamau N, Danielsson H, Callens S, Fransson E, et al. Antibiotic use during pregnancy and the risk of preterm birth: a population-based Swedish cohort study. J Antimicrob Chemother 2022; 77(5):1461-7.

^y^Center for Disease Control and Prevention. Social Determinant of Health available at. <https://www.cdc.gov/about/priorities/why-is-addressing-sdoh-important.html>

^z^SDOH, Social Determinant of Health Database available at <https://www.ahrq.gov/sdoh/data-analytics/sdoh-data.html>
