# Supplementary material for: Novel approaches in linkage of data sources to explore the associations between purchase of opioid prescriptions during pregnancy and adverse neonatal outcomes
Source: PLoS One. 2026 Jan 30;21(1):e0340816. doi: 10.1371/journal.pone.0340816 (PMC12857999; doi:10.1371/journal.pone.0340816)
Supplement: S1 Table — (DOCX) [file pone.0340816.s002.docx]

**S1 Table 1.** Comparison of maternal neighborhood characteristics between the study cohorts and sub-cohorts

| Variable | **Opioid Buyers** | | | | **Opioid**  **Non-Buyers**  N=21600  (78.71%) | *P*-value** |
| --- | --- | --- | --- | --- | --- | --- |
|  | Self-Paid  N=880  (15.07%) | Insurance-Only  N=4961  (84.93%) | *P*-value* | Total  N=5841  (21.29%) |  |  |
| ***Social Determinant of Health (SDOH) of Maternal Neighborhoods^a^*** |  |  |  |  |  |  |
| **Economic stability** |  |  |  |  |  |  |
| Median household income in 2016 dollars estimate, mean [SD] | 41622 (11473) | 41888  (11718) | 0.5279 | 41847.67 (11681.06)^b^ | 43215.44  (12483.52)^c^ | **<0.0001** |
| Percentage of households receiving food stamps/SNAP with income below poverty level, median [min, max] | 8.33  [0, 34.10] | 8.30  [0, 35.00] | 0.9748 | 8.31^d^  [0, 35.00] | 8.10^e^  [0, 69.23] | **<0.0001** |
| Percentage of housing units with no vehicle available, median [min, max] | 6.04  [0, 28.48] | 6.10  [0, 48.15] | 0.1631 | 6.09^d^  [0, 48.15] | 5.99^e^  [0, 48.39] | **<0.0001** |
| **Health care access and quality** |  |  |  |  |  |  |
| Percentage of population with any Medicaid/means-tested, public health coverage median [min, max] | 20.61  [3.41, 47.60] | 20.49  [0, 51.24] | 0.9643 | 20.54  [0, 51.24] | 19.66  [0, 59.52] | **<0.0001** |
| Percentage of population who are uninsured, median [min, max] | 13.76  [0, 41.11] | 13.84  [0, 57.14] | 0.8916 | 13.84  [0, 57.14] | 13.54  [0, 57.14] | **<0.0001** |
| Distance in miles to the nearest emergency room calculated using population weighted ZIP centroids, median [min, max] | 1.77  [0.13, 29.36] | 1.95  [0.04, 29.36] | 0.5476 | 1.91  [0.04, 29.36] | 1.95^f^  [0.13, 29.36] | 0.6999 |
| Distance in miles to the nearest clinic calculated using population weighted ZIP centroids, median [min, max] | 2.85  [0.13, 21.42] | 2.85  [0.10, 26.56] | 0.8766 | 2.85^d^  [0.10, 26.56] | 2.79^f^  [0.07, 26.56] | 0.6542 |
| **Education access and quality** |  |  |  |  |  |  |
| Percentage of population with less than high school education aged 25 years and over, median [min, max] | 15.34  [0, 44.42] | 15.25  [0, 65.63] | 0.5092 | 15.25  [0, 65.63] | 14.87  [0, 64.47] | **<0.0001** |
| Percentage of population with high school education aged 25 years and over, median [min, max] | 35.51  [10.13, 68.57] | 35.3  [0, 75.68] | 0.6135 | 35.33  [0, 75.68] | 34.78  [0, 94.39] | **<0.0001** |
| **Neighborhood and built environment** |  |  |  |  |  |  |
| Percentage of housing units lacking complete plumbing facilities, median [min, max] | 2.48  [0, 40.30] | 2.46  [0, 34.96] | 0.8347 | 2.46^d^  [0, 40.3] | 2.28^e^  [0, 61.47] | **0.0010** |
| Percentage of housing units built before 1979, median [min, max] | 46.79  [8.98, 83.10] | 46.52  [0, 100] | 0.5019 | 46.58^d^  [0, 100] | 45.52^e^  [0, 100] | **<0.0001** |
| **Social and community context** |  |  |  |  |  |  |
| Average household size, mean (SD) | 2.55 (0.23) | 2.54 (0.22) | 0.7639 | 2.54^d^ (0.22) | 2.54^e^ (0.22) | 0.7991 |
| Percentage of population that is foreign-born, median [min, max] | 3.6  [0, 27.87] | 3.45  [0, 27.87] | 0.1618 | 3.55^d^  [0, 27.87] | 3.60^e^  [0, 27.87] | 0.5614 |
| Percentage of limited English-speaking household, median [min, max] | 1.03  [0, 16.83] | 0.92  [0, 20.18] | 0.3558 | 0.93^d^  [0, 20.18] | 0.94^e^  [0, 16.83] | 0.9406 |
| Percentage of householders who are White alone, median [min, max] | 85.12  [12.60, 100] | 83.52  [2.56, 100] | **0.0217** | 83.90^d^  [2.56, 100] | 84.87^e^  [2.56, 100] | **0.0005** |
| Percentage of householders who are Black or African American alone, median [min, max] | 5.30  [0, 86.96] | 8.58  [0, 97.44] | **0.0048** | 7.53^d^  [0, 97.44] | 6.66^e^  [0, 97.44] | **0.0003** |

**Abbreviations by alphabetical order**: max, maximum; min, minimum; SD, standard deviation; SNAP, Supplemental Nutrition Assistance Program; ZIP, Zone improvement Plan.

^a^Variables were selected from the SDOH database to reflect the five domains of social determinants of maternal neighborhood;^b^Data available for only 5828 pregnancies; ^c^Data available for only 21558 pregnancies; ^d^Data available for only 5840 pregnancies; ^e^Data available for only 21598 pregnancies; ^f^Data available for only 21589 pregnancies.

**P*-value for contrast of opioid analgesic self-paid buyers versus insurance-only buyers ;***P*-value for contrast of all opioid analgesic buyers versus non-buyers.
