# Supplementary material for: Novel approaches in linkage of data sources to explore the associations between purchase of opioid prescriptions during pregnancy and adverse neonatal outcomes
Source: PLoS One. 2026 Jan 30;21(1):e0340816. doi: 10.1371/journal.pone.0340816 (PMC12857999; doi:10.1371/journal.pone.0340816)
Supplement: S2 Table — (DOCX) [file pone.0340816.s003.docx]

**S2 Table**. **Summary of results of models evaluating the association between study covariates and neonatal outcomes**

| **^a^Model** | **Comparison among groups** | **NICU admission (yes)** | **LBW (yes)** | **PT (yes)** | **Birth weight z-score** | **NOWS (yes)** |
| --- | --- | --- | --- | --- | --- | --- |
|  |  | AOR (95% CI) | AOR (95% CI) | AOR (95% CI) | Adjusted mean difference  (95% CI) | AOR (95% CI) |
| Model  1 | Opioid buyers vs. non-buyers | 1.26 (1.13-1.41)***** | 1.28 (1.15-1.42)***** | 1.31 (1.19-1.44)***** | -0.0612 (-0.0877; -0.0348)****** | 4.45 (3.22-6.15)****** |
|  | Opioid insurance-only buyers vs. non-buyers | 1.23 (1.09-1.38)**** | 1.25 (1.12-1.40)**** | 1.27 (1.15-1.40)**** | -0.0585 (-0.0869; -0.0302)****** | 3.81 (2.69-5.42)****** |
|  | Opioid self-paid buyers vs. non-buyers | 1.47 (1.16-1.86)**** | 1.44 (1.14-1.80)**** | 1.52 (1.24-1.87)**** | -0.0766 (-0.1377; -0.0154)*** | 8.10  (4.96- 3.22)****** |
|  | Opioid self-paid vs. insurance-only buyers | 0.83 (0.65-1.07) | 0.87 (0.68-1.11) | 0.85 (0.67-1.04) | -0.0181 (-0.0832; 0.0471) | 2.12 (1.28-3.52)**** |
| Model 2 | Opioid buyers vs. non-buyers | 1.23 (1.10-1.37)**** | 1.23 (1.10-1.36)**** | 1.28 (1.16-1.41)***** | -0.0368 (-0.0628; -0.0108)***** | 3.77 (2.71-5.24)****** |
|  | Opioid insurance-only buyers vs. non-buyers | 1.19 (1.06-1.34)**** | 1.20 (1.07-1.34)**** | 1.24 (1.12-1.38)***** | -0.0364 (-0.0641; -0.0087)**** | 3.35 (2.34-4.80)****** |
|  | Opioid self-paid buyers vs. non-buyers | 1.42 (1.12-1.80)**** | 1.38 (1.09-1.74)**** | 1.49 (1.21-1.84)**** | -0.0393 (-0.0987; 0.0202) | 5.74 (3.49-9.44)****** |
|  | Opioid self-paid vs. insurance-only buyers | 0.84 (0.65-1.08) | 0.87 (0.68-1.11) | 0.83 (0.67-1.04) | -0.0029 (-0.0659; 0.0602) | 1.72 (1.03-2.85)*** |
| Model 3 | Opioid buyers vs. non-buyers | 1.23 (1.10-1.37)**** | 1.13 (1.01-1.26)**** | 1.28 (1.16-1.41)***** | -0.0366 (-0.0626; -0.0106)**** | 3.82 (2.74-5.32)****** |
|  | Opioid insurance-only buyers vs. non-buyers | 1.20 (1.06-1.35)**** | 1.10 (0.99-1.24) | 1.24 (1.12-1.37)***** | -0.0361 (-0.0638; -0.0084)*** | 3.39 (2.37-4.87)****** |
|  | Opioid self-paid buyers vs. non-buyers | 1.42 (1.12-1.80)**** | 1.28 (1.03-1.60)*** | 1.49 (1.21-1.84)**** | -0.0391 (-0.0985; 0.0203) | 5.83 (3.54-9.60)****** |
|  | Opioid self-paid vs. insurance-only buyers | 0.84 (0.66-1.08) | 0.86 (0.68-1.09) | 0.83 (0.66-1.04) | -0.0030 (-0.0660; 0.0600) | 1.72 (1.03-2.85)*** |
| Model 4 | Opioid buyers vs. non-buyers | 1.04 (0.92-1.17) | 1.08 (0.96-1.21) | 1.13 (1.02-1.26)*** | -0.0166 (-0.0432; 0.0100) | 2.26 (1.58-3.23)****** |
|  | Opioid insurance-only buyers vs. non-buyers | 1.02 (0.90-1.16) | 1.06 (0.93-1.20) | 1.10 (0.99-1.24) | -0.0189 (-0.0470; 0.0092) | 2.09 (1.42-3.07)****** |
|  | Opioid self-paid buyers vs. non-buyers | 1.11 (0.86-0.43) | 1.16 (0.91-1.48) | 1.28 (1.03-1.60)*** | -0.0029 (-0.0624; 0.0566) | 3.02 (1.79-5.09)****** |
|  | Opioid self-paid vs. insurance-only buyers | 0.92 (0.71-1.20) | 0.91 (0.71-1.18) | 0.86 (0.68-1.09) | 0.0160 (-0.0462; 0.0783) | 1.44 (0.86-2.42) |
| Model 5 | Opioid buyers vs. non-buyers | 1.06 (0.93-1.20) | 1.11 (0.99-1.25) | 1.14 (1.02-1.27)*** | -0.0192 (-0.0464; 0.0080) | 2.19 (1.51-3.18)****** |
|  | Opioid insurance-only buyers vs. non-buyers | 1.05 (0.92-1.20) | 1.11 (0.98-1.25) | 1.12 (0.99-1.25) | -0.0214 (-0.0500; 0.0073) | 2.04 (1.37-3.03)***** |
|  | Opioid self-paid buyers vs. non-buyers | 1.10 (0.85-1.42) | 1.17 (0.91-1.49) | 1.25 (0.99-1.57) | -0.0062 (-0.0661; 0.0536) | 2.88 (1.68-4.91)***** |
|  | Opioid self-paid vs. insurance-only buyers | 0.96 (0.73-1.25) | 0.95 (0.73-1.22) | 0.89 (0.71-1.13) | 0.0151 (-0.0472; 0.0775) | 1.41 (0.84-2.37) |
| Model 6 | Opioid buyers vs. non-buyers | 1.06 (0.93-1.20) | 1.11 (0.99-1.25) | 1.13 (1.02-1.26)*** | -0.0186 (-0.0459; 0.0086) | 2.20 (1.51-3.22)****** |
|  | Opioid insurance-only buyers vs. non-buyers | 1.05 (0.92-1.20) | 1.10 (0.97-1.25) | 1.11 (0.99-1.25) | -0.0207 (-0.0495; 0.0080) | 2.06 (1.36-3.10)***** |
|  | Opioid self-paid buyers vs. non-buyers | 1.09 (0.84-1.40) | 1.16 (0.90-1.49) | 1.25 (0.99-1.57) | -0.0062 (-0.0661; 0.0537) | 2.83 (1.67-4.79)***** |
|  | Opioid self-paid vs. insurance-only buyers | 0.97 (0.74-1.27) | 0.95 (0.73-1.23) | 0.89 (0.70-1.13) | 0.0145 (-0.0479; 0.0770) | 1.37 (0.81-2.32) |
| Model 7 | Opioid buyers vs. non-buyers | 1.07 (0.95-1.22) | 1.13 (0.99-1.27) | 1.14 (1.02-1.28)*** | -0.0231 (-0.0507; 0.0045) | 2.13 (1.44-3.14)***** |
|  | Opioid insurance-only buyers vs. non-buyers | 1.07 (0.93-1.22) | 1.12 (0.98-1.27) | 1.12 (0.99-1.26) | -0.0253 (-0.0544; 0.0037) | 1.98 (1.30-3.01)**** |
|  | Opioid self-paid buyers vs. non-buyers | 1.10 (0.85-1.43) | 1.18 (0.92-1.51) | 1.26 (1.00-1.56)*** | -0.0102 (-0.0703; 0.0499) | 2.75 (1.61-4.72)***** |
|  | Opioid self-paid vs. insurance-only buyers | 0.97 (0.74-1.26) | 0.94 (0.73-1.22) | 0.88 (0.70-1.12) | 0.0152 (-0.0473; 0.0776) | 0.71 (0.43-1.21) |
| Model 8 | Opioid buyers vs. non-buyers | 1.07 (0.94-1.22) | 1.13 (0.99-1.27) | 1.14 (1.02-1.27)*** | -0.0239 (-0.0514; 0.0037) | 2.10 (1.42-3.11)***** |
|  | Opioid insurance-only buyers vs. non-buyers | 1.06 (0.93-1.22) | 1.12 (0.98-1.27) | 1.11 (0.99-1.25) | -0.0261 (-0.0551; 0.0029) | 1.97 (1.29-3.01)**** |
|  | Opioid self-paid buyers vs. non-buyers | 1.10 (0.85-1.43) | 1.17 (0.92-1.50) | 1.26 (1.01-1.58)*** | -0.0107 (-0.0708; 0.0494) | 2.63 (1.54-4.50)***** |
|  | Opioid self-paid vs. insurance-only buyers | 0.97 (0.74-1.26) | 0.95 (0.74-1.23) | 0.88 (0.70-1.12) | 0.0154 (-0.0470; 0.0779) | 1.34 (0.79-2.26) |

^a^Models specifications: Model 1-Unadjusted model, no maternal variables included; Model 2-Adjusted for evaluated maternal demographic variables; Model 3-Adjusted for evaluated maternal demographic characteristics and rurality of area of maternal residence; Model 4-Adjusted for evaluated maternal demographic variables, rurality of area of maternal residence, and evaluated maternal clinical diagnoses (excluding any diagnoses of painful conditions); Model 5-Adjusted for evaluated maternal demographic variables, rurality of area of maternal residence, and evaluated maternal clinical variables (including diagnoses of painful conditions though no diagnoses of painful symptoms); Model 6-Adjusted for evaluated maternal demographic variables, rurality of area of maternal residence, and all evaluated maternal clinical variables (including diagnoses of painful conditions though no diagnoses of painful symptoms) and maternal purchase of pharmacological agents (other than opiate analgesic) during pregnancy; Model 7-Adjusted for evaluated maternal demographic variables, rurality of area of maternal residence, and all evaluated maternal clinical variables including diagnoses of painful conditions and painful symptoms, and maternal purchase of other pharmacological agents (other than opiate analgesic); We evaluated the effects of painful conditions and symptoms in sequential models, as painful symptoms could potentially lie on the causal pathway between painful conditions and opiate purchase and conditioning on this variable may distort the observed relationship between opioid purchases and outcomes; Model 8- Fully adjusted model: adjusted for maternal demographic characteristics, rurality of maternal residence, clinical variables (including diagnoses of painful conditions and painful symptoms), maternal purchase of pharmacologic agents other than opioid analgesics, and neighborhood-level social determinants of health.

Abbreviations (by alphabetical order): AOR: adjusted odds ratio; LBW: low birth weight; NICU: Neonatal Intensive Care Unit; NOWS: neonatal opioid withdrawal syndrome; PT: preterm.

Significance levels: **P* <0.05; ***P* < 0.01; ****P* <0 .001; *****P* <0.0001.
