# Supplementary material for: Novel approaches in linkage of data sources to explore the associations between purchase of opioid prescriptions during pregnancy and adverse neonatal outcomes
Source: PLoS One. 2026 Jan 30;21(1):e0340816. doi: 10.1371/journal.pone.0340816 (PMC12857999; doi:10.1371/journal.pone.0340816)
Supplement: S3 Table — (DOCX) [file pone.0340816.s004.docx]

**S3 Table. Summary of results of fully adjusted models evaluating association between study covariates and neonatal outcomes**

| **Characteristics** | **NICU Admission (yes)** | | **LBW (yes)** | | **PT (yes)** | | **^a^Birth weight z-score** | | **NOWS (yes)** | |
| --- | --- | --- | --- | --- | --- | --- | --- | --- | --- | --- |
|  | AOR (95% CI) | *P*-value | AOR (95% CI) | *P*-value | AOR (95% CI) | *P*-value | Adjusted mean difference  (95% CI) | *P*-value | AOR (95% CI) | *P*-value |
| Opioid buyers vs. non-buyers | 1.07 (0.94-1.22) | 0.2807 | 1.13 (0.99-1.27) | 0.0807 | 1.14 (1.02-1.27) | **0.0243** | -0.0239 (-0.0514; 0.0037) | 0.0898 | 2.10 (1.42-3.11) | **0.0002** |
| ***Maternal demographic characteristics*** |  | |  | |  | |  | |  | |
| Age  <20  ≥20–<30  ≥30 | 1.00 (0.84-1.19)  Reference  1.09 (0.97-1.22) | 0.9913  0.1597 | 1.00 (0.85-1.18)  Reference  1.06 (0.94-1.19) | 0.9664  0.3364 | 1.03 (0.88-1.20)  Reference  1.18 (1.06-1.31) | 0.7129  **0.0020** | -0.0292 (-0.0645; 0.0062)  Reference  0.0448 (0.0188; 0.0709) | 0.1056  **0.0007** | 0.25 (0.09-0.70)  Reference  1.73 (1.20-2.51) | **0.0083**  **0.0035** |
| Race/ethnicity  Non-Hispanic White  Non-Hispanic Black  Others | Reference  1.17 (1.01-1.37)  1.11 (0.91-1.34) | **0.0377**  0.2867 | Reference  1.96 (1.70-2.26)  1.38 (1.14-1.68) | **<.0.0001**  **0.0012** | Reference  1.29 (1.14-1.48)  1.05 (0.88-1.25) | **0.0001**  0.5775 | Reference  -0.3345 (-0.3675; -0.3014)  -0.1647 (-0.2067; -0.1226) | **<0.0001**  **<0.0001** | Reference  0.55 (0.31-0.98)  0.30 (0.10-0.89) | **0.0416**  **0.0307** |
| Marital status  No  Yes | Reference  0.90 (0.79-1.02) | 0.0976 | Reference  0.87 (0.77-0.98) | **0.0229** | Reference  0.89 (0.80-1.00) | **0.0491** | Reference  0.0792 (0.0527; 0.1056) | **<0.0001** | Reference  0.95 (0.63-1.41) | 0.7825 |
| Prenatal care  No  Yes  Unknown | Reference  0.46 (0.36-0.61)  0.58 (0.42-0.79) | **<0.0001**  **0.0006** | Reference  0.48 (0.38-0.63)  1.10 (0.82-1.47 | **<.0.0001**  0.5180 | Reference  0.39 (0.31-0.49)  0.94 (0.72-1.23) | **<0.0001**  0.6707 | Reference  -0.1012 (-0.1745; -0.0279)  -0.0570 (-0.1384; 0.0245) | **0.0068**  0.1705 | Reference  0.26 (0.14-0.48)  0.49 (0.21-1.13) | **<.0.0001**  0.0951 |
| Eligibility for WIC  No  Yes | Reference  0.93 (0.84-1.04) | 0.2119 | Reference  0.90 (0.81-1.00) | 0.0527 | Reference  0.90 (0.81-0.99) | **0.0346** | Reference  -0.0032 (-0.0272; 0.0207) | 0.7919 | Reference  0.83 (0.57-2.00) | 0.3169 |
| Pre-pregnancy BMI  <=18  19-24  25-29  >=30 | 1.14 (0.94-1.38)  Reference  0.93 (0.81-1.07)  0.98 (0.86-1.11) | 0.1883  0.3059  0.7149 | 1.14 (0.95-1.36)  Reference  0.72 (0.63-0.82)  0.63 (0.56-0.72) | 0.1549  **<0.0001**  **<0.0001** | 1.27 (1.08-1.50)  Reference  0.83 (0.74-0.94)  0.77 (0.68-0.86) | **0.0048**  **0.0033**  **<0.0001** | -0.0539 (-0.0950; -0.0128)  Reference  0.0959 (0.0685; 0.1233)  0.2219 (0.1952; 0.2486) | **0.0102**  **<0.0001**  **<0.0001** | 1.26 (0.71-2.23)  Reference  0.96 (0.62-1.50)  0.81 (0.52-1.26) | 0.4232  0.8746  0.3456 |
| Geographic location of maternal residence  North  Central  South | Reference  0.96 (0.83-1.11)  0.93 (0.78-1.11) | 0.5618  0.4354 | Reference  0.94 (0.81-1.09)  1.17 (1.00-1.36) | 0.4167  **0.0462** | Reference  0.83 (0.72-0.95)  1.23 (1.07-1.42) | **0.0069**  **0.0041** | Reference  0.0421 (0.0106; 0.0736)  0.0136 (-0.0226; 0.0499) | **0.0088**  0.4612 | Reference  0.47 (0.28-0.81)  0.97 (0.56-1.69) | **0.0067**  0.9149 |
| Level of education  Less or equal to high school  More than high school | Reference  0.95 (0.85-1.07) | 0.4202 | Reference  0.78 (0.70-0.87) | **<.0.0001** | Reference  0.82 (0.74-0.91) | **0.0001** | Reference  0.0718 (0.0476; 0.0960) | **<0.0001** | Reference  0.96 (0.66-1.39) | 0.8313 |
| Insurance type  Medicaid  Private insurance  Other | Reference  1.07 (0.94-1.20)  0.49 (0.39-0.62) | 0.3032  **<0.0001** | Reference  0.96 (0.85-1.08)  1.05 (0.88-1.25) | 0.5096  0.6136 | Reference  1.06 (0.95-1.18)  1.17 (0.99-1.37) | 0.3199  0.0601 | Reference  0.0628 (0.0374; 0.0882)  0.0455 (0.0044; 0.0865) | **<0.0001**  **0.0301** | Reference  0.74 (0.49-1.12)  0.77 (0.41-1.46) | 0.1495  0.4219 |
| ***Rurality of maternal area of residence*** |  | |  | |  | |  | |  | |
| Primary Rural-Urban Commuting Area Code 2010  Metropolitan  Micropolitan  Small town  Rural | Reference  0.54 (0.46-0.64)  0.72 (0.60-0.87)  0.69 (0.52-0.91) | **<0.0001**  **0.0006**  **0.0081** | Reference  1.01 (0.87-1.17)  0.99 (0.84-1.18)  0.78 (0.58-1.03) | 0.9319  0.9283  0.0812 | Reference  1.07 (0.94-1.22)  1.02 (0.87-1.20)  0.77 (0.60-1.00) | 0.3122  0.7832  0.0528 | Reference  0.0149 (-0.0171; 0.0469)  -0.0427 (-0.0818; -0.0036)  -0.0218 (-0.0768; 0.0333) | 0.3609  **0.0322**  0.4388 | Reference  0.49 (0.30-0.80)  0.47 (0.25-0.90)  1.04 (0.48-2.24) | **0.0046**  **0.0227**  0.9248 |
| ***Maternal medical diagnosis*** |  | |  | |  | |  | |  | |
| Number of mental diagnoses  0  1  ≥2 | Reference  1.13 (0.97-1.33)  1.31 (1.13-1.52) | 0.1165  **0.0004** | Reference  0.98 (0.84-1.14)  1.14 (0.99-1.33) | 0.7646  0.0808 | Reference  0.98 (0.85-1.13)  1.15 (1.00-1.32) | 0.7819  0.0558 | Reference  -0.0136 (-0.0497; 0.0224)  0.0375 (-0.0007; 0.0757) | 0.4590  0.0543 | Reference  2.18 (1.37-3.45)  2.80 (1.85-4.25) | **0.0010**  **<0.0001** |
| Alcohol or substance related disorders  No  Yes | Reference  1.27 (1.09-1.47) | **0.0019** | Reference  1.18 (1.03-1.37) | **0.0196** | Reference  1.07 (0.93-1.23) | 0.3376 | Reference  -0.0973 (-0.1333; -0.0612) | **<0.0001** | Reference  3.52 (2.26-5.50) | **<0.0001** |
| Smoking  No  Yes | Reference  1.15 (1.01-1.31) | **0.0318** | Reference  1.46 (1.29-1.64) | **<0.0001** | Reference  1.12 (1.00-1.25) | 0.0599 | Reference  -0.1841 (-0.2121; -0.1562) | **<0.0001** | Reference  1.57 (0.94-2.62) | 0.0838 |
| Diabetes  No  Yes | Reference  1.31 (1.15-1.51) | **<0.0001** | Reference  0.89 (0.76-1.03) | 0.1265 | Reference  1.57 (1.39-1.77) | **<0.0001** | Reference  0.2717 (0.2340; 0.3093) | **<0.0001** | Reference  1.10 (0.67-1.80) | 0.7084 |
| Placental pathologies  No  Yes | Reference  2.62 (2.28-3.02) | **<0.0001** | Reference  3.30 (2.89-3.77) | **<0.0001** | Reference  2.65 (2.33-3.02) | **<0.0001** | Reference  -0.0828 (-0.1307; -0.0350) | **0.0007** | Reference  2.10 (1.34-3.29) | **0.0013** |
| Prior cesarean section  No  Yes | Reference  1.12 (0.99-1.27) | 0.0609 | Reference  0.97 (0.86-1.09) | 0.6218 | Reference  0.94 (0.85-1.05) | 0.3025 | Reference  0.0855 (0.0575; 0.1135) | **<0.0001** | Reference  0.82 (0.54-1.24) | 0.3427 |
| Pregnancy induced hypertension or hypertension  No  Yes | Reference  1.36 (1.17-1.58) | **<0.0001** | Reference  1.31 (1.13-1.51) | **0.0003** | Reference  1.40 (1.23-1.59) | **<0.0001** | Reference  0.0344 (-0.0082; 0.0771) | 0.1137 | Reference  1.28 (0.78-2.11) | 0.3254 |
| Preeclampsia  No  Yes | Reference  1.96 (1.71-2.27) | **<0.0001** | Reference  2.52 (2.21-2.88) | **<0.0001** | Reference  2.66 (2.36-2.99) | **<0.0001** | Reference  -0.0553 (-0.0968; -0.0137) | **0.0092** | Reference  1.14 (0.66-1.98) | 0.6443 |
| Renal diseases  No  Yes | Reference  1.49 (1.09-2.05) | **0.0136** | Reference  1.28 (0.91-1.82) | 0.1611 | Reference  1.63 (1.21-2.20) | **0.0013** | Reference  -0.0134 (-0.1178; 0.0910) | 0.8017 | Reference  0.87 (0.31-2.38) | 0.7770 |
| Cardiac diseases  No  Yes | Reference  1.66 (1.31-2.09) | **<0.0001** | Reference  1.17 (0.92-1.51) | 0.2131 | Reference  1.12 (0.90-1.41) | 0.3344 | Reference  -0.0284 (-0.0952; 0.0384) | 0.4050 | Reference  1.15 (0.52-2.55) | 0.7351 |
| Infectious diseases  No  Yes | Reference  1.55 (1.34-1.79) | **<0.0001** | Reference  1.15 (0.99-1.33) | 0.0653 | Reference  1.34 (1.17-1.53) | **<0.0001** | Reference  0.0810 (0.0438; 0.1182) | **<0.0001** | Reference  1.87 (1.25-2.81) | **0.0024** |
| Sepsis or shock  No  Yes | Reference  1.39 (0.78-2.46) | 0.2647 | Reference  1.42 (0.78-2.58) | 0.2464 | Reference  1.39 (0.81-2.37) | 0.2325 | Reference  -0.1244 (-0.2937; 0.0450) | 0.1500 | Reference  1.04 (0.31-3.47) | 0.9512 |
| Gastrointestinal diseases  No  Yes | Reference  1.02 (0.90-1.15) | 0.8118 | Reference  1.03 (0.91-1.16) | 0.6637 | Reference  1.03 (0.92-1.15) | 0.5742 | Reference  -0.0216 (-0.0497; 0.0065) | 0.1324 | Reference  0.97 (0.67-1.40) | 0.8743 |
| Pulmonary pathologies  No  Yes | Reference  0.98 (0.94-1.32) | 0.2040 | Reference  1.05 (0.88-1.24) | 0.6189 | Reference  1.02 (0.87-1.20) | 0.8169 | Reference  -0.0230 (-0.0650; 0.0191) | 0.2844 | Reference  1.21 (0.74-1.95) | 0.4488 |
| Hematological diseases  No  Yes | Reference  0.85 (0.75-0.97) | **0.0156** | Reference  0.87 (0.77-0.99) | **0.0333** | Reference  0.95 (0.84-1.06) | 0.3338 | Reference  0.0302 (0.0023; 0.0580) | **0.0338** | Reference  0.95 (0.62-1.44) | 0.8077 |
| Other maternal diagnoses  No  Yes | Reference  0.96 (0.68-1.35) | 0.7982 | Reference  1.11 (0.79-1.57) | 0.5473 | Reference  0.92 (0.67-1.27) | 0.6281 | Reference  -0.1104 (-0.1912; -0.0296) | **0.0074** | Reference  1.64 (0.72-3.74) | 0.2357 |
| ***Maternal painful conditions*** |  | |  | |  | |  | |  | |
| Arthropathies  No  Yes | Reference  0.96 (0.82-1.11) | 0.5560 | Reference  0.89 (0.77-1.03) | 0.1103 | Reference  0.94 (0.83-1.08) | 0.3936 | Reference  0.0230 (-0.0103; 0.0563) | 0.1763 | Reference  1.11 (0.73-1.68) | 0.6399 |
| Migraines and/or headache  No  Yes | Reference  0.91 (0.79-1.04) | 0.1680 | Reference  0.90 (0.78-1.02) | 0.1066 | Reference  0.92 (0.83-1.06) | 0.2961 | Reference  -0.0245 (-0.0549; 0.0059) | 0.1146 | Reference  0.78 (0.51-1.20) | 0.2518 |
| Neuropathies  No  Yes | Reference  1.02 (0.81-1.28) | 0.8714 | Reference  1.13 (0.90-1.42) | 0.2868 | Reference  1.15 (0.94-1.41) | 0.1659 | Reference  -0.0057 (-0.0627; 0.0513) | 0.8451 | Reference  0.82 (0.44-1.52) | 0.5313 |
| Injuries  No  Yes | Reference  1.02 (0.89-1.16) | 0.7997 | Reference  0.95 (0.83-1.08) | 0.4013 | Reference  1.02 (0.91-1.14) | 0.7575 | Reference  -0.0018 (-0.0317; 0.0281) | 0.9038 | Reference  1.30 (0.89-1.91) | 0.1773 |
| ***Maternal procedures*** |  |  |  |  |  |  |  |  |  |  |
| Any procedure  No  Yes | Reference  1.57 (1.36-1.82) | <0.0001 | Reference  1.61 (1.40-1.85) | **<0.0001** | Reference  1.61 (1.40-1.82) | **<0.0001** | Reference  0.0178 (-0.0206; 0.0562) | 0.3640 | Reference  1.19 (0.75-1.90) | 0.4686 |
| **Maternal exposure to other pharmacological agents** |  |  |  |  |  |  |  |  |  |  |
| Ten days increase in count of days of Buprenorphine scripts | 0.92 (0.67-1.25) | 0.5950 | 1.06 (0.81-1.38) | 0.6734 | 0.92 (0.56-1.51) | 0.7535 | -0.0582 (-0.1095; -0.0068) | **0.0264** | 1.70 (0.89-3.26) | 0.1092 |
| Antibiotics  No  Yes | Reference  0.84 (0.73-0.98) | **0.0258** | Reference  0.85 (0.74-0.99) | **0.0338** | Reference  0.85 (0.75-0.97) | **0.0125** | Reference  -0.0082 (-0.0381; 0.0217) | 0.5911 | Reference  0.72 (0.42-1.26) | 0.2526 |
| Thyroid therapeutic agents  No  Yes | Reference  0.67 (0.39-1.14) | 0.1408 | Reference  0.70 (0.39-1.26) | 0.2326 | Reference  0.68 (0.41-1.11) | 0.1223 | Reference  0.0182 (-0.0917; 0.1280) | 0.7459 | Reference  1.62 (0.42-6.20) | 0.4851 |
| Cardiovascular therapeutic agents  No  Yes | Reference  1.54 (1.19-2.00) | **0.0011** | Reference  1.68 (1.31-2.16) | **<0.0001** | Reference  1.77 (1.42-2.22) | **<0.0001** | Reference  0.0125 (-0.0624; 0.0874) | 0.7432 | Reference  1.66 (0.78-3.55) | 0.1886 |
| Benzodiazepines  No  Yes | Reference  0.87 (0.62-1.23) | 0.4327 | Reference  0.96 (0.68-1.34) | 0.7924 | Reference  1.04 (0.77-1.39) | 0.8074 | Reference  0.0189 (-0.0612; 0.0991) | 0.6437 | Reference  1.62 (0.78-3.36) | 0.1951 |
| Selective Serotonin Reuptake inhibitor (SSRI)  No  Yes | Reference  0.97 (0.75-1.25) | 0.8081 | Reference  1.30 (1.01-1.65) | **0.0377** | Reference  0.99 (0.79-1.24) | 0.9127 | Reference  -0.0363 (-0.0932; 0.0206) | 0.2115 | Reference  0.67 (0.33-1.38) | 0.2812 |
| Tricyclic antidepressants other than SSRI  No  Yes | Reference  1.18 (0.86-1.63) | 0.3134 | Reference  0.85 (0.60-1.20) | 0.3454 | Reference  0.97 (0.73-1.29) | 0.8388 | Reference  0.0374 (-0.0376; 0.1123) | 0.3283 | Reference  0.70 (0.31-1.63) | 0.4168 |
| Antipsychotic therapeutic agents  No  Yes | Reference  1.41 (0.82-2.42) | 0.2181 | Reference  0.57 (0.28-1.15) | 0.1138 | Reference  0.88 (0.51-1.52) | 0.6492 | Reference  -0.0198 (-0.1631; 0.1235) | 0.7869 | Reference  0.88 (0.24-3.23) | 0.8477 |
| Barbiturate or seizure therapeutic agents  No  Yes | Reference  0.86 (0.61-1.22) | 0.4050 | Reference  0.87 (0.61-1.25) | 0.4539 | Reference  1.26 (0.95-1.67) | 0.1149 | Reference  -0.0029 (-0.0784; 0.0725) | 0.9390 | Reference  0.57 (0.22-1.45) | 0.2370 |
| Amphetamine therapeutic agents  No  Yes | Reference  0.87 (0.49-1.53) | 0.6244 | Reference  1.14 (0.67-1.94) | 0.6376 | Reference  0.90 (0.54-1.47) | 0.6645 | Reference  -0.0239 (-0.1343; 0.0866) | 0.6719 | Reference  2.05 (0.61-6.80) | 0.2400 |
| Gamma-aminobutyric acid (GABA) therapeutic agents  No  Yes | Reference  1.55 (0.45-5.38) | 0.4895 | Reference  1.62 (0.39-6.77) | 0.5064 | Reference  0.76 (0.17-3.39) | 0.7139 | Reference  -0.1781 (-0.5209; 0.1647) | 0.3085 | Reference  7.47 (1.84-30.25) | **0.0048** |
| ***Maternal painful symptoms*** |  | |  | |  | |  | |  | |
| Number of painful diagnoses  0  1  ≥2 | Reference  1.03 (0.91-1.16)  0.91 (0.78-1.07) | 0.6191  0.2461 | Reference  1.04 (0.92-1.17)  0.93 (0.80-1.08) | 0.5320  0.3530 | Reference  1.04 (0.94-1.16)  0.96 (0.83-1.10) | 0.4479  0.5411 | Reference  0.0247 (-0.0014; 0.0508)  0.0412 (0.0076; 0.0748) | 0.0640  **0.0162** | Reference  1.39 (0.85-2.27)  1.38 (0.82-2.34) | 0.1883  0.2123 |
| ***Social determinant of health database variables*** |  |  |  |  |  |  |  |  |  |  |
| One thousand dollars increase in median household income | 1.00 (0.99-1.01) | 0.0519 | 0.99 (0.98-1.01) | 0.4620 | 0.99 (0.98-1.01) | 0.3310 | 0.0000 (-0.0002; 0.0002) | 0.8492 | 1.00 (0.99-1.01) | 0.8745 |
| Ten percent increase in housing units with no vehicle available | 0.81 (0.66-1.01) | 0.0534 | 0.87 (0.73-1.04) | 0.1319 | 0.93 (0.79-1.10) | 0.4060 | 0.0450 (0.0046; 0.0853) | 0.0291 | 0.83 (0.46-1.47) | 0.5167 |
| Ten percent increase in population who are uninsured | 1.17 (0.99-1.37) | 0.0590 | 1.05 (0.97-1.15) | 0.2112 | 1.16 (1.01-1.33) | 0.0391 | 0.0147 (-0.0199; 0.0493) | 0.4065 | 1.39 (0.87-2.23) | 0.1660 |
| Increase in distance by 10 miles to the nearest clinic calculated using population weighted ZIP centroids | 1.00 (0.98-1.01) | 0.6556 | 1.18 (1.02-1.37) | **0.0240** | 0.99 (0.98-1.01) | 0.2609 | 0.0004 (-0.0023; 0.0030) | 0.7873 | 1.01 (0.97-1.05) | 0.6149 |
| Ten percent increase of population with high school education aged 25 years and over | 0.94 (0.86-1.03) | 0.1995 | 0.99 (0.98-1.01) | 0.1487 | 1.02 (0.94-1.10) | 0.6844 | -0.0074 (-0.0261; 0.0114) | 0.4401 | 1.17 (0.87-1.57) | 0.2870 |
| Ten percent increase of housing units lacking complete plumbing facilities | 0.97 (0.80-1.17) | 0.7253 | 1.11 (0.94-1.32) | 0.2249 | 0.97 (0.83-1.15) | 0.7522 | 0.0183 (-0.0266; 0.0632) | 0.4246 | 1.26 (0.72-2.20) | 0.4207 |
| Ten percent increase of housing units built before 1979 | 1.00 (0.99-1.04) | 0.9950 | 0.96 (0.93-1.01) | 0.0839 | 0.97 (0.93-1.01) | 0.0722 | -0.0013 (-0.0101; 0.0076) | 0.7820 | 1.06 (0.91-1.24) | 0.4768 |
| Average increase in household size by 1 | 0.93 (0.69-1.26) | 0.6510 | 0.69 (0.52-0.91) | **0.0095** | 0.94 (0.74-1.22) | 0.6433 | 0.0667 (0.0049; 0.1284) | **0.0343** | 0.98 (0.36-2.67) | 0.9729 |
| Ten percent increase of householders who are Black or African American alone | 0.97 (0.93-1.01) | 0.1425 | 1.01 (0.97-1.04) | 0.7665 | 1.00 (0.97-1.04) | 0.7915 | -0.0167 (-0.0256; -0.0078) | **0.0003** | 0.96 (0.85-1.10) | 0.5774 |

Abbreviations: AOR, adjusted odds ratio; CI, confidence interval; NICU, neonatal intensive care unit; LBW, low birth weight; PT, preterm birth; NOWS, neonatal opioid withdrawal syndrome.

Models were estimated using generalized estimating equations with an exchangeable working correlation to account for clustering of pregnancies within mothers. Logistic links were used for binary outcomes and an identity link for the continuous outcome (birth weight z-score). All models adjusted for demographic, clinical, and neighborhood covariates.

^a^Adjusted mean difference represents the effect estimate for birth weight z-score derived from the GEE model with an identity link.
